# A systematic review of natural language processing applied to radiology reports

**DOI:** 10.1186/s12911-021-01533-7

**Published:** 2021-06-03

**Authors:** Arlene Casey, Emma Davidson, Michael Poon, Hang Dong, Daniel Duma, Andreas Grivas, Claire Grover, Víctor Suárez-Paniagua, Richard Tobin, William Whiteley, Honghan Wu, Beatrice Alex

**Affiliations:** 1 School of Literatures, Languages and Cultures (LLC), University of Edinburgh, Edinburgh, Scotland; 2 Centre for Clinical Brain Sciences, University of Edinburgh, Edinburgh, Scotland; 3 Centre for Medical Informatics, Usher Institute of Population Health Sciences and Informatics, University of Edinburgh, Edinburgh, Scotland; 4 Health Data Research UK, London, UK; 5 Institute for Language, Cognition and Computation, School of informatics, University of Edinburgh, Edinburgh, Scotland; 6 Nuffield Department of Population Health, University of Oxford, Oxford, UK; 7 Institute of Health Informatics, University College London, London, UK; 8 Edinburgh Futures Institute, University of Edinburgh, Edinburgh, Scotland

**Keywords:** Natural language processing, Radiology, Systematic review

## Abstract

**Background:**

Natural language processing (NLP) has a significant role in advancing healthcare and has been found to be key in extracting structured information from radiology reports. Understanding recent developments in NLP application to radiology is of significance but recent reviews on this are limited. This study systematically assesses and quantifies recent literature in NLP applied to radiology reports.

**Methods:**

We conduct an automated literature search yielding 4836 results using automated filtering, metadata enriching steps and citation search combined with manual review. Our analysis is based on 21 variables including radiology characteristics, NLP methodology, performance, study, and clinical application characteristics.

**Results:**

We present a comprehensive analysis of the 164 publications retrieved with publications in 2019 almost triple those in 2015. Each publication is categorised into one of 6 clinical application categories. Deep learning use increases in the period but conventional machine learning approaches are still prevalent. Deep learning remains challenged when data is scarce and there is little evidence of adoption into clinical practice. Despite 17% of studies reporting greater than 0.85 F1 scores, it is hard to comparatively evaluate these approaches given that most of them use different datasets. Only 14 studies made their data and 15 their code available with 10 externally validating results.

**Conclusions:**

Automated understanding of clinical narratives of the radiology reports has the potential to enhance the healthcare process and we show that research in this field continues to grow. Reproducibility and explainability of models are important if the domain is to move applications into clinical use. More could be done to share code enabling validation of methods on different institutional data and to reduce heterogeneity in reporting of study properties allowing inter-study comparisons. Our results have significance for researchers in the field providing a systematic synthesis of existing work to build on, identify gaps, opportunities for collaboration and avoid duplication.

**Supplementary Information:**

The online version contains supplementary material available at 10.1186/s12911-021-01533-7.

## Background

Medical imaging examinations interpreted by radiologists in the form of narrative reports are used to support and confirm diagnosis in clinical practice. Being able to accurately and quickly identify the information stored in radiologists’ narratives has the potential to reduce workloads, support clinicians in their decision processes, triage patients to get urgent care or identify patients for research purposes. However, whilst these reports are generally considered more restricted in vocabulary than other electronic health records (EHR), e.g. clinical notes, it is still difficult to access this efficiently at scale [1]. This is due to the unstructured nature of these reports and Natural Language Processing (NLP) is key to obtaining structured information from radiology reports [2].

NLP applied to radiology reports is shown to be a growing field in earlier reviews [2, 3]. In recent years there has been an even more extensive growth in NLP research in general and in particular deep learning methods which is not seen in the earlier reviews. A more recent review of NLP applied to radiology-related research can be found but this focuses on one NLP technique only, deep learning models [4]. Our paper provides a more comprehensive review comparing and contrasting all NLP methodologies as they are applied to radiology.

It is of significance to understand and synthesise recent developments specific to NLP in the radiology research field as this will assist researchers to gain a broader understanding of the field, provide insight into methods and techniques supporting and promoting new developments in the field. Therefore, we carry out a systematic review of research output on NLP applications in radiology from 2015 onward, thus, allowing for a more up to date analysis of the area. An additional listing of our synthesis of publications detailing their clinical and technical categories can be found in Additional file 1 and per publication properties can be found in Additional file 2. Also different to the existing work, we look at both the clinical application areas NLP is being applied in and consider the trends in NLP methods. We describe and discuss study properties, e.g. data size, performance, annotation details, quantifying these in relation to both the clinical application areas and NLP methods. Having a more detailed understanding of these properties allows us to make recommendations for future NLP research applied to radiology datasets, supporting improvements and progress in this domain.

## Related work

Amongst pre-existing reviews in this area, [2] was the first that was both specific to NLP on radiology reports and systematic in methodology. Their literature search identified 67 studies published in the period up to October 2014. They examined the NLP methods used, summarised their performance and extracted the studies’ clinical applications, which they assigned to five broad categories delineating their purpose. Since Pons et al.’s paper, several reviews have emerged with the broader remit of NLP applied to electronic health data, which includes radiology reports. [5] conducted a systematic review of NLP systems with a specific focus on coding free text into clinical terminologies and structured data capture. The systematic review by [6] specifically examined machine learning approaches to NLP (2015–2019) in more general clinical text data, and a further methodical review was carried out by [7] to synthesise literature on deep learning in clinical NLP (up to April 2019) although the did not follow the PRISMA guideline completely. With radiology reports as their particular focus, [3] published, the same year as Pons et al.’s review, an instructive narrative review outlining the fundamentals of NLP techniques applied in radiology. More recently, [4] published a systematic review focused on deep learning radiology-related research. They identified 10 relevant papers in their search (up to September 2019) and examined their deep learning models, comparing these with traditional NLP models and also considered their clinical applications but did not employ a specific categorisation. We build on this corpus of related work, and most specifically Pons et al.’s work. In our initial synthesis of clinical applications we adopt their application categories and further expand upon these to reflect the nature of subsequent literature captured in our work. Additionally, we quantify and compare properties of the studies reviewed and provide a series of recommendations for future NLP research applied to radiology datasets in order to promote improvements and progress in this domain.

## Methods

Our methodology followed the Preferred Reporting Items for Systematic Reviews and Meta-Analysis (PRISMA) [8], and the protocol is registered on protocols.io.

### Eligibility for literature inclusion and search strategy

We included studies using NLP on radiology reports of any imaging modality and anatomical region for NLP technical development, clinical support, or epidemiological research. Exclusion criteria included: (1) language not English; (2) wrong publication type (e.g., case reports, reviews, conference abstracts, comments, patents, or editorials) (2) published before 2015; (3) uses radiology images only (no NLP); (4) not radiology reports; (5) no NLP results; (6) year out of range; (7) duplicate, already in the list of publications retrieved; (8) not available in full text.

We used Publish or Perish [9], a citation retrieval and analysis software program, to search Google Scholar. Google Scholar has a similar coverage to other databases [10] and is easier to integrate into search pipelines. We conducted an initial pilot search following the process described here, but the search terms were too specific and restricted the number of publications. For example, we experimented with using specific terms used within medical imaging such at CT, MRI. Thirty-seven papers were found during the pilot search but the same papers also appeared in our final search. We use the following search query restricted to research articles published in English between January 2015 and October 2019. (“radiology” OR “radiologist”) AND (“natural language” OR “text mining” OR “information extraction” OR “document classification” OR “word2vec”) NOT patent. We automated the addition of publication metadata and applied filtering to remove irrelevant publications. These automated steps are described in Tables 1 and 2.

**Table 1:** Metadata enriching steps undertaken for each publication

Metadata enriching steps	
1. Match the paper with its DOI via the Crossref API [11]	
2. If DOI matched, check Semantic Scholar for metadata/abstract [12]	
3. If no DOI match and no abstract, search PubMed for abstract	
4. Search arXiv [13] (for a pre-print)	
5. If no PDF link, search Unpaywall for available open access versions [14]	
6. If PDF but no separate abstract via Semantics Scholar/PubMed, extract abstract from the PDF

**Table 2:** Automated filtering steps to remove irrelevant publications

Automated filtering steps	
1. Document language is English	
2. Word ’patent’ in title or URL	
3. Year of publication out of range (<2015)	
4. The words ’review’ or ’overview’ in the title, ’this review’ in the abstract	
5. Image keywords in title or abstract with no NLP terminology in abstract	
6. No radiology keywords in title or abstract	
7. No NLP terminology in abstract	

In addition to query search, another method to find papers is to conduct a citation search [15]. The citation search compiled a list of publications that cite the Pons et al. review and the articles cited in the Pons’ review. To do this, we use a snowballing method [16] to follow the forward citation branch for each publication in this list, i.e. finding every article that cites the publications in our list. The branching factor here is large, so we filter at every stage and automatically add metadata. One hundred and seventy-one papers were identified as part of the snowball citation search and of these 84 were in the final 164 papers.

### Manual review of literature

Four reviewers (three NLP researchers [AG,DD and HD] and one epidemiologist [MTCP]) independently screened all titles and abstracts with the Rayyan online platform and discussed disagreements. Fleiss’ kappa [17] agreement between reviewers was 0.70, indicating substantial agreement [18]. After this screening process, each full-text article was reviewed by a team of eight (six NLP researchers and two epidemiologists) and double reviewed by a NLP researcher. We resolved any discrepancies by discussion in regular meetings.

### Data extraction for analysis

We extracted data on: primary clinical application and technical objective, data source(s), study period, radiology report language, anatomical region, imaging modality, disease area, dataset size, annotated set size, training/validation/test set size, external validation performed, domain expert used, number of annotators, inter-annotator agreement, NLP technique(s) used, best-reported results (recall, precision and F1 score), availability of dataset, and availability of code.

## Results

**Fig. 1:**
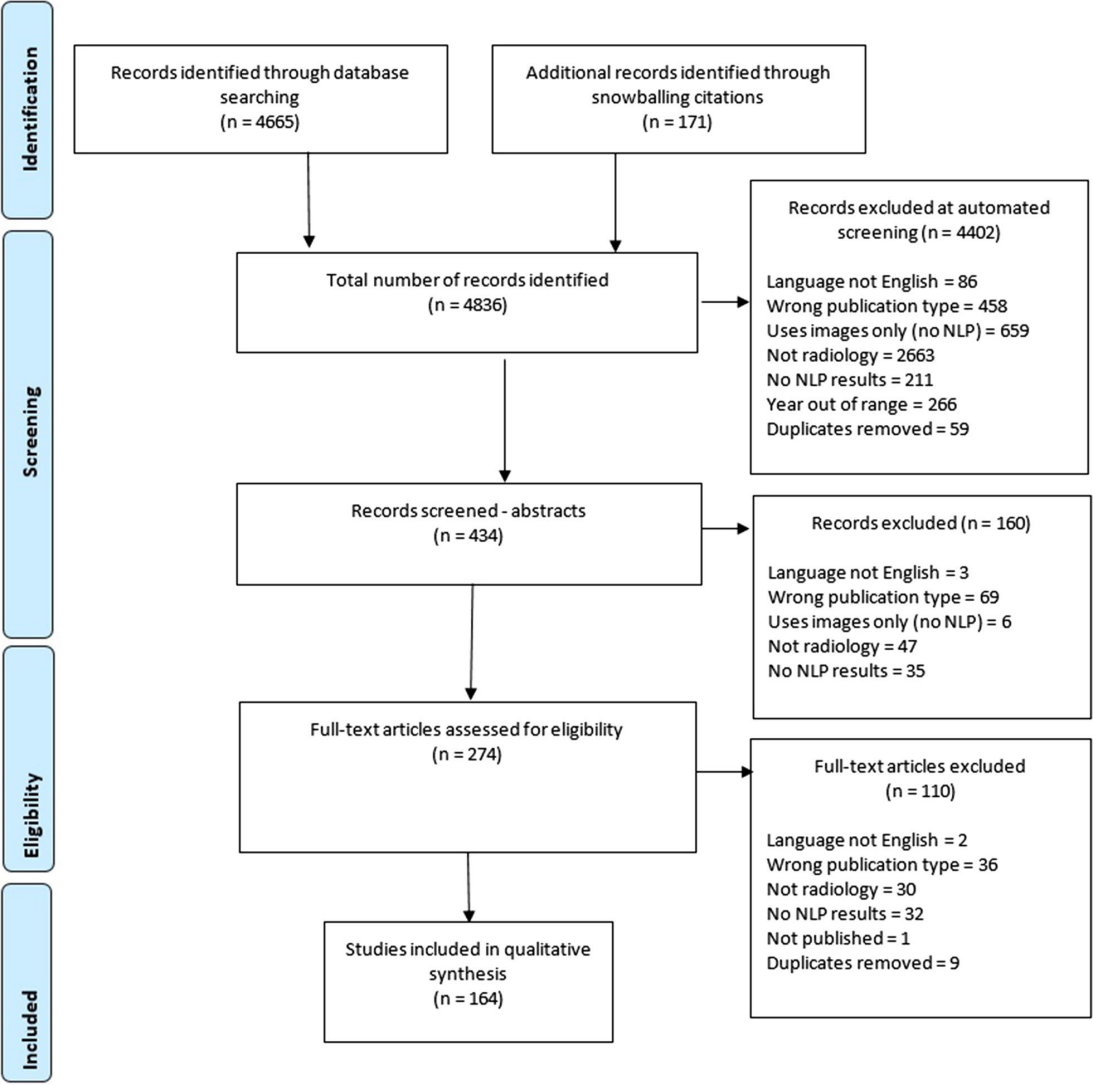
PRISMA diagram for search publication retrieval

The literature search yielded 4836 possibly relevant publications from which our automated exclusion process removed 4,402, and during both our screening processes, 270 were removed, leaving 164 publications. See Fig. 1 for details of exclusions at each step.

### General characteristics

**Fig. 2:**
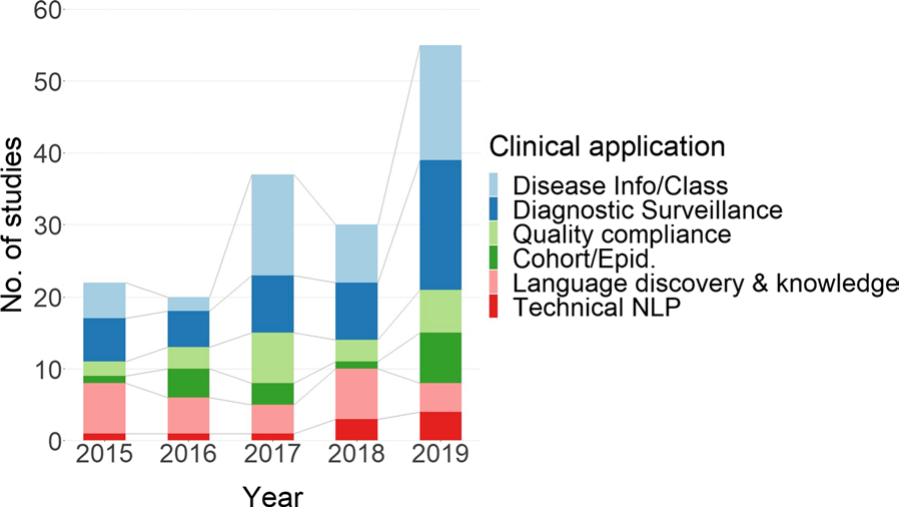
Clinical application of publication by year

2015 and 2016 saw similar numbers of publications retrieved (22 and 21 respectively) with the volume increasing almost three-fold in 2019 (55), noting 2019 only covers 10 months (Fig. 2). Imaging modality (Table 3) varied considerably and 46 studies used reports from multiple modalities. Of studies focusing on a single modality, the most featured were CT scans (38) followed by MRI (16), X-Ray (8), Mammogram (5) and Ultrasound (4). Forty-seven studies did not specifying scan modality. For the study samples (Table 4), 33 papers specified that they used consecutive patient images, 38 used non-consecutive image sampling and 93 did not clearly specify their sampling strategy. The anatomical regions for scans varied (Table 5) with mixed being the highest followed by Thorax and Head/neck. Disease categories are presented in Table 6 with the largest disease category being Oncology. The majority of reports were in English (141) and a small number in other languages e.g., Chinese (5), Spanish (4), German (3) (Table 7). Additional file 2, CSV format, provides a breakdown of the information in Tables 3, 4, 5, 6 and 7 per publication.

**Table 3:** Scan modality

Scan modality	No. studies
Multiple modalities	46
MRI	16
CT	38
X-Ray	8
Mammogram	5
Ultrasound	4
Not specified	47
Total	164

**Table 4:** Image sampling method

Sampling method	No. studies
Consecutive images	33
Non-consecutive images	38
Not specified	93
Total	164

**Table 5:** Anatomical region scanned

Anatomical region	No. studies
Mixed	43
Thorax	32
Head/neck	25
Abdomen	15
Breast	15
Extremities	9
Spine	5
Other	1
Unspecified	19
Total	164

**Table 6:** Disease category

Disease category	No. studies
Not specific disease related	40
Oncology	39
Various	20
Musculoskeletal	10
Cerebrovascular	13
Other	13
Respiratory	10
Trauma	7
Cardiovascular	6
Gastrointestinal	3
Hepatobiliary	2
Genitourinary	1
Total	164

**Table 7:** Radiology report language

Report language	No. studies
English	141
Chinese	5
Spanish	4
German	3
Italian	2
French	2
Hebrew	1
Polish	1
Brazilian Portuguese	1
Unspecified	4
Total	164

**Table 8:** Clinical application category by technical objective

Application category	Information extraction	Report/sentence	Lexicon/ ontology	Clustering
	(n = 73)	Classification (n = 81)	Discovery (n = 9)	(n = 1)
Disease information & Classification	15	31	-	-
Diagnostic surveillance	28	17	-	-
Quality compliance	5	15	–	–
Cohort-Epid.	6	10	–	–
Language discovery & knowledge	13	4	9	1
Technical NLP	6	4	–	–

### Clinical application categories

In synthesis of the literature each publication was classified by the primary clinical purpose. Pons’ work in 2016 categorised publications into 5 broad categories: Diagnostic Surveillance, Cohort Building for Epidemiological Studies, Query-based Case Retrieval, Quality Assessment of Radiological Practice and Clinical Support Services. We found some changes in this categorisation schema and our categorisation consisted of six categories: *Diagnostic Surveillance, Disease information and classification, Quality Compliance, Cohort/Epidemiology, Language Discovery and Knowledge Structure, Technical NLP*. The main difference is we found no evidence for a category of *Clinical Support Services* which described applications that had been integrated into the workflow to assist. Despite the increase in the number of publications, very few were in clinical use with more focus on the category of *Disease Information and Classification*. We describe each clinical application area in more detail below and where applicable how our categories differ from the earlier findings. A listing of all publications and their corresponding clinical application and technical category can be found in Additional file 1, MS Word format, and in Additional file 2 in CSV format. Table 8 shows the clinical application category by the technical classification and Fig. 2 shows the breakdown of clinical application category by publication year. There were more publications in 2019 compared with 2015 for all categories except Language Discovery & Knowledge Structure, which fell by $\approx$ 25% (Fig. 2).

#### Diagnostic surveillance

A large proportion of studies in this category focused on extracting disease information for patient or disease surveillance e.g. investigating tumour characteristics [19, 20]; changes over time [21] and worsening/progression or improvement/response to treatment [22, 23]; identifying correct anatomical labels [24]; organ measurements and temporality [25]. Studies also investigated pairing measurements between reports [26] and linking reports to monitoring changes through providing an integrated view of consecutive examinations [27]. Studies focused specifically on breast imaging findings investigating aspects, such as BI-RADS MRI descriptors (shape, size, margin) and final assessment categories (benign, malignant etc.) e.g., [28–33]. Studies focused on tumour information e.g., for liver [34] and hepatocellular carcinoma (HPC) [35, 36] and one study on extracting information relevant for structuring subdural haematoma characteristics in reports [37].

Studies in this category also investigated incidental findings including on lung imaging [38–40], with [38] additionally extracting the nodule size; for trauma patients [41]; and looking for silent brain infarction and white matter disease [42]. Other studies focused on prioritising/triaging reports, detecting follow-up recommendations, and linking a follow-up exam to the initial recommendation report, or bio-surveillance of infectious conditions, such as invasive mould disease.

#### Disease information and classification

*Disease Information and Classification* publications use reports to identify information that may be aggregated according to classification systems. These publications focused solely on classifying a disease occurrence or extracting information about a disease with no focus on the overall clinical application. This category was not found in Pons’ work. Methods considered a range of conditions including intracranial haemorrhage [43, 44], aneurysms [45], brain metastases [46], ischaemic stroke [47, 48], and several classified on types and severity of conditions e.g., [46, 49–52]. Studies focused on breast imaging considered aspects such as predicting lesion malignancy from BI-RADS descriptors [53], breast cancer subtypes [54], and extracting or inferring BI-RADS categories, such as [55, 56]. Two studies focused on abdominal images and hepatocellular carcinoma (HCC) staging and CLIP scoring. Chest imaging reports were used to detect pulmonary embolism e.g., [57–59], bacterial pneumonia [60], and Lungs-RADS categories [61]. Functional imaging was also included, such as echocardiograms, extracting measurements to evaluate heart failure, including left ventricular ejection fractions (LVEF) [62]. Other studies investigated classification of fractures [63, 64] and abnormalities [65] and the prediction of ICD codes from imaging reports [66].

#### Language discovery and knowledge structure

*Language Discovery and Knowledge Structure* publications investigate the structure of language in reports and how this might be optimised to facilitate decision support and communication. Pons et al. reported on applications of *Query-based retrieval* which has similarities to *Language Discovery and Knowledge Structure* but it is not the same. Their category contains studies that retrieve cases and conditions that are not predefined and in some instances could be used for research purposes or are motivated for educational purposes. Our category is broader and encompasses papers that investigated different aspects of language including variability, complexity simplification and normalising to support extraction and classification tasks.

Studies focus on exploring lexicon coverage and methods to support language simplification for patients looking at sources, such as the consumer health vocabulary [67] and the French lexical network (JDM) [68]. Other works studied the variability and complexity of report language comparing free-text and structured reports and radiologists. Also investigated was how ontologies and lexicons could be combined with other NLP methods to represent knowledge that can support clinicians. This work included improving report reading efficiency [69]; finding similar reports [70]; normalising phrases to support classification and extraction tasks, such as entity recognition in Spanish reports [71]; imputing semantic classes for labelling [72], supporting search [73] or to discover semantic relations [74].

#### Quality and compliance

*Quality and Compliance* publications use reports to assess the quality and safety of practice and reports similar to Pons’ category. Works considered how patient indications for scans adhered to guidance e.g., [75–80] or protocol selection [81–85] or the impact of guideline changes on practice, such as [86]. Also investigated was diagnostic utilisation and yield, based on clinicians or on patients, which can be useful for hospital planning and for clinicians to study their work patterns e.g. [87]. Other studies in this category looked at specific aspects of quality, such as, classification for long bone fractures to support quality improvement in paediatric medicine [88], automatic identification of reports that have critical findings for auditing purposes [89], deriving a query-based quality measure to compare structured and free-text report variability [90], and [91] who describe a method to fix errors in gender or laterality in a report.

#### Cohort and epidemiology

This category is similar to Pons’ earlier review but we treated the studies in this category differently attempting to differentiate which papers described methods for creating cohorts for research purposes, and those which also reported the outcomes of an epidemiological analysis. Ten studies use NLP to create specific cohorts for research purposes and six reported the performance of their tools. Out of these papers, the majority (n = 8) created cohorts for specific medical conditions including fatty liver disease [92, 93] hepatocellular cancer [94], ureteric stones [95], vertebral fracture [96], traumatic brain injury [97, 98], and leptomeningeal disease secondary to metastatic breast cancer [99]. Five papers identified cohorts focused on particular radiology findings including ground glass opacities (GGO) [100], cerebral microbleeds (CMB) [101], pulmonary nodules [102, 103], changes in the spine correlated to back pain [1] and identifying radiological evidence of people having suffered a fall. One paper focused on identifying abnormalities of specific anatomical regions of the ear within an audiology imaging database [104] and another paper aimed to create a cohort of people with any rare disease (within existing ontologies - Orphanet Rare Disease Ontology ORDO and Radiology Gamuts Ontology RGO). Lastly, one paper took a different approach of screening reports to create a cohort of people with contraindications for MRI, seeking to prevent iatrogenic events [105]. Amongst the epidemiology studies there were various analytical aims, but they primarily focused on estimating the prevalence or incidence of conditions or imaging findings and looking for associations of these conditions/findings with specific population demographics, associated factors or comorbidities. The focus of one study differed in that it applied NLP to healthcare evaluation, investigating the association of palliative care consultations and measures of high-quality end-of-life (EOL) care [99].

#### Technical NLP

This category is for publications that have a primary technical aim that is not focused on radiology report outcome, e.g. detecting negation in reports, spelling correction [106], fact checking [107, 108] methods for sample selection, crowd source annotation [109]. This category did not occur in Pons’ earlier review.

### NLP methods in use

NLP methods capture the different techniques an author applied broken down into rules, machine learning methods, deep learning, ontologies, lexicons and word embeddings. We discriminate machine learning from deep learning, using the former to represent traditional machine learning methods.

**Fig. 3:**
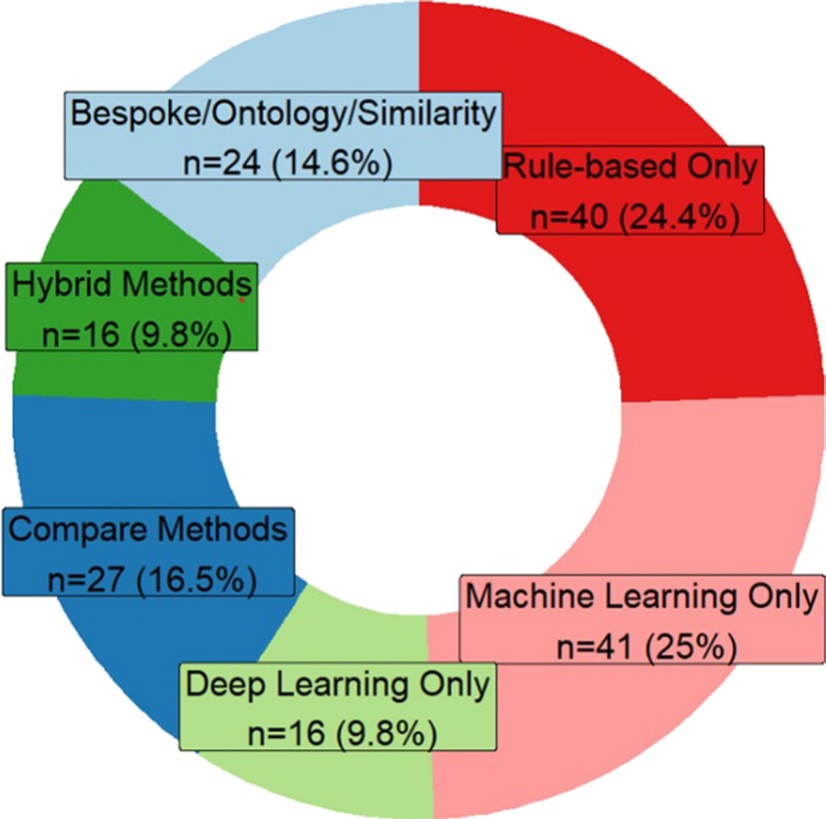
NLP method breakdown

**Fig. 4:**
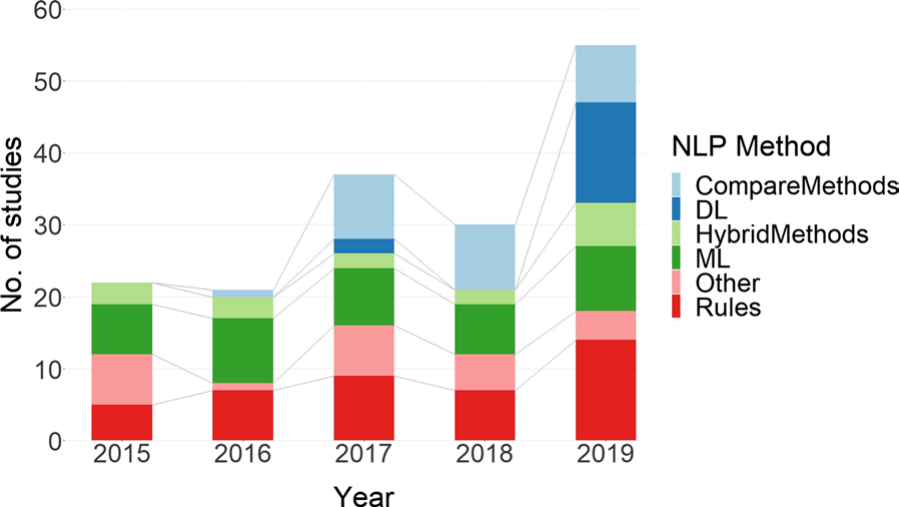
NLP method by year

Over half of the studies only applied one type of NLP method and just over a quarter of the studies compared or combined methods in hybrid approaches. The remaining studies either used a bespoke proprietary system or focus on building ontologies or similarity measures (Fig. 3). Rule-based method use remains almost constant across the period, whereas use of machine learning decreases and deep learning methods rises, from five publications in 2017 to twenty-four publications in 2019 (Fig. 4).

**Table 9:** Breakdown of NLP method

ML (n = 74)	No studies	Deep learning (n = 36)	No studies
SVM	34	RNN variants	14
Logistic regression	23	CNN	10
Random forest	18	Other	5
Naïve Bayes	17	Compare CNN, RNN	4
Maximum entropy	7	Combine CNN + RNN	3
Decision trees	4		

A variety of machine classifier algorithms were used, with SVM and Logistic Regression being the most common (Table 9). Recurrent Neural Networks (RNN) variants were the most common type of deep learning architectures. RNN methods were split between long short-term memory (LSTM) and bidirectional-LSTM (Bi-LSTM), bi-directional gated recurrent unit (Bi-GRU), and standard RNN approaches. Four of these studies additionally added a Conditional Random Field (CRF) for the final label generation step. Convolutional Neural Networks (CNN) were the second most common architecture explored. Eight studies additionally used an attention mechanism as part of their deep learning architecture. Other neural approaches included feed-forward neural networks, fully connected neural networks and a proprietary neural system IBM Watson [82] and Snorkel [110]. Several studies proposed combined architectures, such as [31, 111].

### NLP method features

Most rule-based and machine classifying approaches used features based on bag-of-words, part-of-speech, term frequency, and phrases with only two studies alternatively using word embeddings. Three studies use feature engineering with deep learning rather than word embeddings. Thirty-three studies use domain-knowledge to support building features for their methods, such as developing lexicons or selecting terms and phrases. Comparison of embedding methods is difficult as many studies did not describe their embedding method. Of those that did, Word2Vec [112] was the most popular (n = 19), followed by GLOVE embeddings [113] (n = 6), FastText [114] (n = 3), ELMo [115] (n = 1) and BERT [116] (n = 1). Ontologies or lexicon look-ups are used in 100 studies; however, even though publications increase over the period in real terms, 20% fewer studies employ the use of ontologies or lexicons in 2019 compared to 2015. The most widely used resources were UMLS [117] (n = 15), Radlex [118] (n = 20), SNOMED-CT [119] (n = 14). Most studies used these as features for normalising words and phrases for classification, but this was mainly those using rule-based or machine learning classifiers with only six studies using ontologies as input to their deep learning architecture. Three of those investigated how existing ontologies can be combined with word embeddings to create domain-specific mappings, with authors pointing to this avoiding the need for large amounts of annotated data. Other approaches looked to extend existing medical resources using a frequent phrases approach, e.g. [120]. Works also used the derived concepts and relations visualising these to support activities, such as report reading and report querying (e.g. [121, 122])

### Annotation and inter-annotator agreement

Eighty-nine studies used at least two annotators, 75 did not specify any annotation details, and only one study used a single annotator. Whilst 69 studies use a domain expert for annotation (a clinician or radiologist) only 56 studies report the inter-annotator agreement. Some studies mention annotation but do not report on agreement or annotators. Inter-annotator agreement values for Kappa range from 0.43 to perfect agreement at 1. Whilst most studies reported agreement by Cohen’s Kappa [123] some reported precision, and percent agreement. Studies reported annotation data sizes differently, e.g., on the sentence or patient level. Studies also considered ground truth labels from coding schemes such as ICD or BI-RADS categories as annotated data. Of studies which detailed human annotation at the radiology report level, only 45 specified inter-annotator agreement and/or the number of annotators. Annotated report numbers for these studies varies with 15 papers having annotated less than 500, 12 having annotated between 500 and less than 1000, 15 between 1000 and less than 3000, and 3 between 4000 and 8,288 reports. Additional file 2 gives all annotation size information on a per publication basis in CSV format.

### Data sources and availability

Only 14 studies reported that their data is available, and 15 studies reported that their code is available. Most studies sourced their data from medical institutions, a number of studies did not specify where their data was from, and some studies used publicly available datasets: MIMIC-III (n = 5), MIMIC-II (n = 1), MIMIC-CXR (n = 1); Radcore (n = 5) or STRIDE (n = 2). Four studies used combined electronic health records such as clinical notes or pathology reports.

**Table 10:** NLP Method by data size properties, minimum data size, maximum data size and median value, studies reporting in numbers of radiology reports

NLP method	Min size	Max size	Median
Compare methods	513	2,167,445	2,845
Hybrid methods	40	34,926	918
Deep learning (Only)	120	1,567,581	5000
Machine learning (Only)	101	2,977,739	2531
Rules (only)	31	10,000,000	8000
Other	25	12,377,743	10,000

**Table 11:** Grouped data size and number of studies in each group, only for studies reporting in numbers of radiology reports

Data size group	No. studies (%)
<200	9 (6.7)
200 < 500	6 (4.4)
500 < 1000	18 (13.3)
1000 < 2000	17 (12.6)
2000 < 5000	17 (12.6)
5000 < 10,000	12 (8.9)
10,000+	53 (39.3)
Unspecified	3 (2.2)

**Table 12:** Studies reporting on total data size used and details on training set size, validation set size, test set size and annotation set size

Dataset type	No. of studies	Comments
Total dataset size	151	5
Training set size	129	
Validation set size	52	27 report size, 25 report k-fold cross validation
Test set size	81	
Annotation set size	97	

Reporting on total data size differed across studies with some not giving exact data sizes but percentages and others reporting numbers of sentences, reports, patients, or a mixture of these. Where an author was not clear on the type of data they were reporting on, or on the size, we marked this as unspecified. Thirteen studies did not report on total data size. Data size summaries for those reporting at the radiology report level is n = 135 or 82.32% of the studies (Table 10). The biggest variation of data size by NLP Method is in studies that apply other methods or are rule-based. Machine learning also varies in size; however, the median value is lower compared to rule-based methods. The median value for deep learning is considerably higher at 5000 reports compared to machine learning or those that compare or create hybrid methods. Of the studies reporting on radiology reports numbers, 39.3% used over 10,000 reports and this increases to over 48% using more than 5000 reports. However, a small number of studies, 14%, are using comparatively low numbers of radiology reports, less than 500 (Table 11).

### NLP performance and evaluation measures

**Fig. 5:**
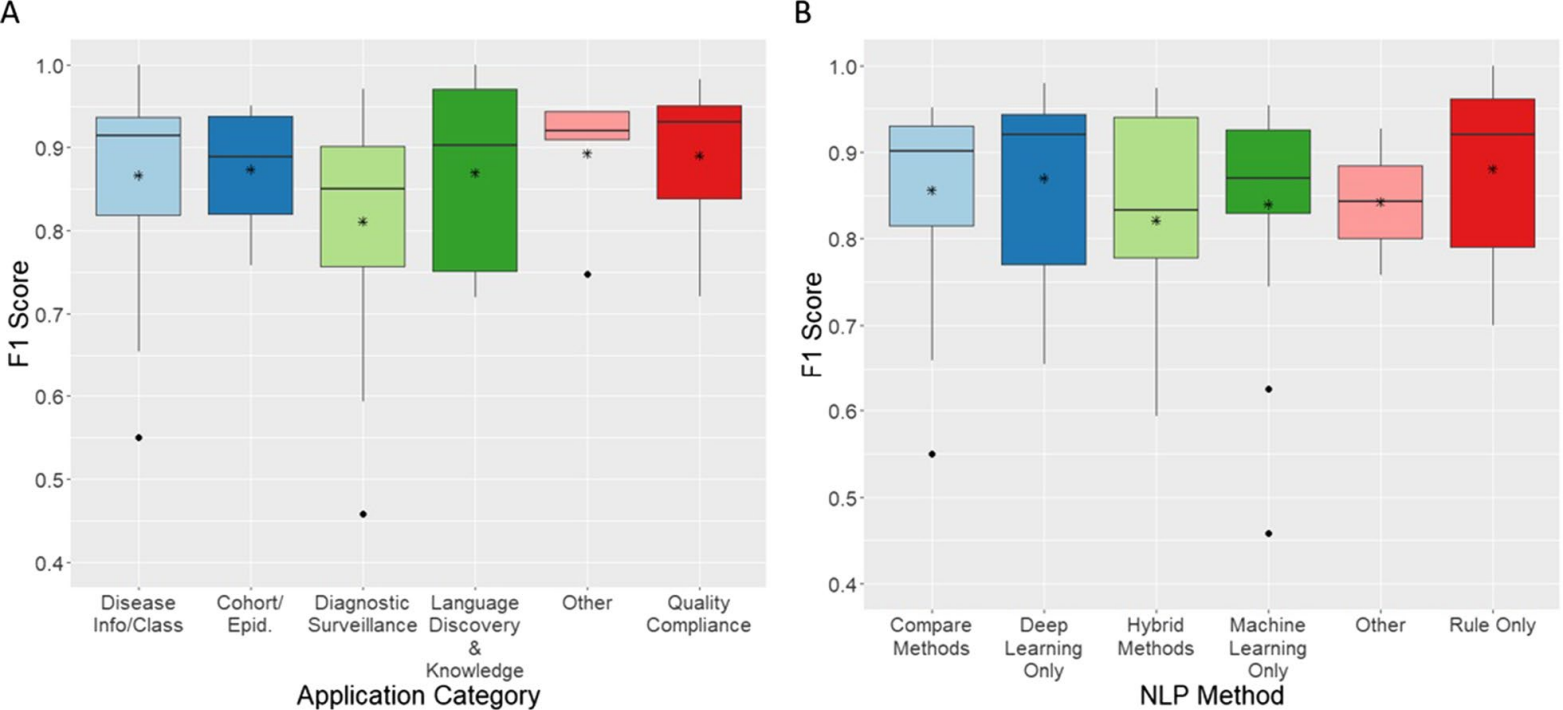
Application Category and NLP Method, Mean and Median Summaries. Mean value is indicated by a vertical bar, the box shows error bars and the asterisk is the median value

Performance metrics applied for evaluation of methods vary widely with authors using precision (positive predictive value (PPV)), recall (sensitivity), specificity, the area under the curve (AUC) or accuracy. We observed a wide variety in evaluation methodology employed concerning test or validation datasets. Different approaches were taken in generating splits for testing and validation, including k-fold cross-validation. Table 12 gives a summary of the number of studies reporting about total data size and splits across train, validation, test, and annotation. This table is for all data types, i.e., reports, sentences, patients or mixed. Eighty-two studies reported on both training and test data splits, of which only 38 studies included a validation set. Only 10 studies validated their algorithm using an external dataset from another institution, another modality, or a different patient population. Additional file 2 gives all data size information on a per publication basis in CSV format. The most widely used metrics for reporting performance were precision (PPV) and recall (sensitivity) reported in 47% of studies. However, even though many studies compared methods and reported on the top-performing method, very few studies carried out significance testing on these comparisons. Issues of heterogeneity make it difficult and unrealistic to compare performance between methods applied, hence, we use summary measures as a broad overview (Fig. 5). Performance reported varies, but both the mean and median values for the F1 score appear higher for methods using rule-based only or deep learning only methods. Whilst differences are less discernible between F1 scores for application areas, *Diagnostic Surveillance* looks on average lower than other categories.

## Discussion and future directions

Our work shows there has been a considerable increase in the number of publications using NLP on radiology reports over the recent time period. Compared to 67 publications retrieved in the earlier review of [2], we retrieved 164 publications. In this section we discuss and offer some insight into the observations and trends of how NLP is being applied to radiology and make some recommendations that may benefit the field going forward.

### Clinical applications and NLP methods in radiology

The clinical applications of the publications is similar to the earlier review of Pons et al. but whilst we observe an increase in research output we also highlight that there appears to be even less focus on clinical application compared to their review. Like many other fields applying NLP the use of deep learning has increased, with RNN architectures being the most popular. This is also observed in a review of NLP in clinical text [7]. However, although deep learning use increases, rules and traditional machine classifiers are still prevalent and often used as baselines to compare deep learning architectures against. One reason for traditional methods remaining popular is their interpretability compared to deep learning models. Understanding the features that drive a model prediction can support decision-making in the clinical domain but the complex layers of non-linear data transformations deep learning is composed of does not easily support transparency [124]. This may also help explain why in synthesis of the literature we observed less focus on discussing clinical application and more emphasis on disease classification or information task only. Advances in interpretability of deep learning models are critical to its adoption in clinical practice.

Other challenges exist for deep learning such as only having access to small or imbalanced datasets. Chen et al. [125] review deep learning methods within healthcare and point to these challenges resulting in poor performance but that these same datasets can perform well with traditional machine learning methods. We found several studies highlight this and when data is scarce or datasets imbalanced, they introduced hybrid approaches of rules and deep learning to improve performance, particularly in the *Diagnostic Surveillance* category. Yang et al. [126] observed rules performing better for some entity types, such as time and size, which are proportionally lower than some of the other entities in their train and test sets; hence they combine a bidirectional-LSTM and CRF with rules for entity recognition. Peng et al. [19] comment that combining rules and the neural architecture complement each other, with deep learning being more balanced between precision and recall, but the rule-based method having higher precision and lower recall. The authors reason that this provides better performance as rules can capture rare disease cases, particularly when multi-class labelling is needed, whilst deep learning architectures perform worse in instances with fewer data points.

In addition to its need for large-scale data, deep learning can be computationally costly. The use of pre-trained models and embeddings may alleviate some of this burden. Pre-trained models often only require fine-tuning, which can reduce computation cost. Language comprehension pre-learned from other tasks can then be inherited from the parent models, meaning fewer domain-specific labelled examples may be needed [127]. This use of pre-trained information also supports generalisability, e.g., [58] show that their model trained on one dataset can generalise to other institutional datasets.

Embedding use has increased which is expected with the application of deep learning approaches but many rule-based and machine classifiers continue to use traditional count-based features, e.g., bag-of-words and n-grams. Recent evidence [128] suggests that the trend to continue to use feature engineering with traditional machine learning methods does produce better performance in radiology reports than using domain-specific word embeddings.

Banerjee et al. [44] found that there was not much difference between a uni-gram approach and a Word2vec embedding, hypothesising this was due to their narrow domain, intracranial haemorrhage. However, the NLP research field has seen a move towards bi-directional encoder representations from transformers (BERT) based embedding models not reflected in our analysis, with only one study using BERT generated embeddings [46]. Embeddings from BERT are thought to be superior as they can deliver better contextual representations and result in improved task performance. Whilst more publications since our review period have used BERT based embeddings with radiology reports e.g. [127, 129] not all outperform traditional methods [130]. Recent evidence shows that embeddings generated by BERT fail to show a generalisable understanding of negation [131], an essential factor in interpreting radiology reports effectively. Specialised BERT models have been introduced such as ClinicalBERT [132] or BlueBERT [129]. BlueBERT has been shown to outperform ClinicalBERT when considering chest radiology [133] but more exploration of the performance gains versus the benefits of generalisability are needed for radiology text.

All NLP models have in common that they need large amounts of labelled data for model training [134]. Several studies [135–137] explored combining word embeddings and ontologies to create domain-specific mappings, and they suggest this can avoid a need for large amounts of annotated data. Additionally, [135, 136] highlight that such combinations could boost coverage and performance compared to more conventional techniques for concept normalisation.

The number of publications using medical lexical knowledge resources is still relatively low, even though a recent trend in the general NLP field is to enhance deep learning with external knowledge [138]. This was also observed by [7], where only 18% of the deep learning studies in their review utilised knowledge resources. Although pre-training supports learning previously known facts it could introduce unwanted bias, hindering performance. The inclusion of domain expertise through resources such as medical lexical knowledge may help reduce this unwanted bias [7]. Exploration of how this domain expertise can be incorporated with deep learning architectures in future could improve the performance when having access to less labelled data.

### Task knowledge

Knowledge about the disease area of interest and how aspects of this disease are linguistically expressed is useful and could promote better performing solutions. Whilst [139] find high variability between radiologists, with metric values (e.g. number of syntactic, clinical terms based on ontology mapping) being significantly greater on free-text than structured reports, [140] who look specifically at anatomical areas find less evidence for variability. Zech et al. [141] suggest that the highly specialised nature of each imaging modality creates different sub-languages and the ability to discover these labels (i.e. disease mentions) reflects the consistency with which labels are referred to. For example, edema is referred to very consistently whereas other labels are not, such as infarction/ischaemic. Understanding the language and the context of entity mentions could help promote novel ideas on how to solve problems more effectively. For example, [35] discuss how the accuracy of predicting malignancy is affected by cues being outside their window of consideration and [142] observe problems of co-reference resolution within a report due to long-range dependencies. Both these studies use traditional NLP approaches, but we observed novel neural architectures being proposed to improve performance in similar tasks specifically capturing long-range context and dependency learning, e.g., [31, 111]. This understanding requires close cooperation of healthcare professionals and data scientists, which is different to some other fields where more disconnection is present [125].

### Study heterogeneity, a need for reporting standards

Most studies reviewed could be described as a proof-of-concept and not trialled in a clinical setting. Pons et al. [2] hypothesised that a lack of clinical application may stem from uncertainty around minimal performance requirements hampering implementations, evidence-based practice requiring justification and transparency of decisions, and the inability to be able to compare to human performance as the human agreement is often an unknown. These hypotheses are still valid, and we see little evidence that these problems are solved.

Human annotation is generally considered the gold standard at measuring human performance, and whilst many studies reported that they used annotated data, overall, reporting was inconsistent. Steps were undertaken to measure inter-annotator agreement (IAA), but in many studies, this was not directly comparable to the evaluation undertaken of the NLP methods. The size of the data being used to draw experimental conclusions from is important and accurate reporting of these measures is essential to ensure reproducibility and comparison in further studies. Reporting on the training, test and validation splits was varied with some studies not giving details and not using held-out validation sets.

Most studies use retrospective data from single institutions but this can lead to a model over-fitting and, thus, not generalising well when applied in a new setting. Overcoming the problem of data availability is challenging due to privacy and ethics concerns, but essential to ensure that performance of models can be investigated across institutions, modalities, and methods. Availability of data would allow for agreed benchmarks to be developed within the field that algorithm improvements can be measured upon. External validation of applied methods was extremely low, although, this is likely due to the availability of external datasets. Making code available would enable researchers to report how external systems perform on their data. However, only 15 studies reported that their code is available. To be able to compare systems there is a need for common datasets to be available to benchmark and compare systems against.

Whilst reported figures in precision and recall generally look high more evidence is needed for accurate comparison to human performance. A wide variety of performance measures were used, with some studies only reporting one measure, e.g., accuracy or F1 scores, with these likely representing the best performance obtained. Individual studies are often not directly comparable for such measures, but none-the-less clarity and consistency in reporting is desirable. Many studies making model comparisons did not carry out any significance testing for these comparisons.

### Progressing NLP in radiology

The value of NLP applied to radiology is clear in that it can support areas such as clinicians in their decision making and reducing workload, add value in terms of automated coding of data, finding missed diagnosis for triage or monitoring quality. However, in recent years labelling disease phenotypes or extracting disease information in reports has been a focus rather than real-world clinical application of NLP within radiology. We believe this is mainly due to the difficulties in accessing data for research purposes. More support is needed to bring clinicians and NLP experts together to promote innovative thinking about how such work can benefit and be trialled in the clinical environment. The challenges in doing so are significant because of the need to work within safe environments to protect patient privacy. In terms of NLP methods, we observe that the general trends of NLP are applied within this research area, but we would emphasise as NLP moves more to deep learning it is particularly important in healthcare to think about how these methods can satisfy explainability. Explainability in artificial intelligence and NLP has become a hot topic in general but it is now also being addressed in the healthcare sector [143, 144]. Methodology used is also impacted by data availability with uncommon diseases often being hard to predict with deep learning as data is scarce. If the practical and methodological challenges on data access, privacy and less data demanding approaches can be met there is much potential to increase the value of NLP within radiology. The sharing of tools, practice, and expertise could also ease the real-world application of NLP within radiology.

To help move the field forward, enable more inter-study comparisons, and increase study reproducibility we make the following recommendations for research studies:

1. Clarity in reporting study properties is required: (a) Data characteristics including size and the type of dataset should be detailed, e.g., the number of reports, sentences, patients, and if patients how many reports per patient. The training, test and validation data split should be evident, as should the source of the data. (b) Annotation characteristics including the methodology to develop the annotation should be reported, e.g., annotation set size, annotator details, how many, expertise. (c) Performance metrics should include a range of metrics: precision, recall, F1, accuracy and not just one overall value.
2. Significance testing should be carried out when a comparison between methods is made.
3. Data and code availability are encouraged. While making data available will often be challenging due to privacy concerns, researchers should make code available to enable inter-study comparisons and external validation of methods.
4. Common datasets should be used to benchmark and compare systems.

### Limitations of study

Publication search is subject to bias in search methods and it is likely that our search strategy did inevitably miss some publications. Whilst trying to be precise and objective during our review process some of the data collected and categorising publications into categories was difficult to agree on and was subjective. For example, many of the publications could have belonged to more than one category. One of the reasons for this was how diverse in structure the content was which was in some ways reflected by the different domains papers were published in. It is also possible that certain keywords were missed in recording data elements due to the reviewers own biases and research experience.

## Conclusions

This paper presents an systematic review of publications using NLP on radiology reports during the period 2015 to October 2019. We show there has been substantial growth in the field particularly in researchers using deep learning methods. Whilst deep learning use has increased, as seen in NLP research in general, it faces challenges of lower performance when data is scarce or when labelled data is unavailable, and is not widely used in clinical practice perhaps due to the difficulties in interpretability of such models. Traditional machine learning and rule-based methods are, therefore, still widely in use. Exploration of domain expertise such as medial lexical knowledge must be explored further to enhance performance when data is scarce. The clinical domain faces challenges due to privacy and ethics in sharing data but overcoming this would enable development of benchmarks to measure algorithm performance and test model robustness across institutions. Common agreed datasets to compare performance of tools against would help support the community in inter-study comparisons and validation of systems. The work we present here has the potential to inform researchers about applications of NLP to radiology and to lead to more reliable and responsible research in the domain.

## Supplementary Information

**Additional file 1.** Publication list with application and technical categories.

**Additional file 2.** Individual properties for every publication.

## Data Availability

All data generated or analysed during this study are included in this published article [and its supplementary information files].

## Abbreviations

NLP: natural language processing
e.g.: example
ICD: international classification of diseases
BI-RADS: Breast Imaging-Reporting and Data System
IAA: inter-annotator agreement
No.: number
UMLS: unified medical language system
ELMo: embeddings from Language Models
BERT: bidirectional encoder representations from transformers
SVM: support vector machine
CNN: convolutional neural network
LSTM: long short-term memory
Bi-LSTM: bi-directional long short-term memory
RU: bi-directional gated recurrent unit
CRF: conditional random field
GLOVE: Global Vectors for Word Representation
