# Supplementary material for: A systematic review of natural language processing applied to radiology reports
Source: BMC Med Inform Decis Mak. 2021 Jun 3;21:179. doi: 10.1186/s12911-021-01533-7 (PMC8176715; doi:10.1186/s12911-021-01533-7)
Supplement: Supplementary file 1 — Additional file 1. Publication list with application and technical categories. [file 12911_2021_1533_MOESM1_ESM.docx]

Title: A Systematic Review of Natural Language Processing Applied to Radiology Reports

Authors: Arlene Casey, Emma Davidson , Michael Poon, Hang Dong, Daniel Duma, Andreas Grivas, Claire Grover, Victor Suarez-Paniagua , Richard Tobin, William Whiteley, Honghan Wu and Beatrice Alex

| **Application Area** | **Sub-Category** | **Technical Task** | **Anatomical Scan Region**  **(Paper Reference Number)** |
| --- | --- | --- | --- |
| **Diagnostic Surveillance (45)**  Using imaging reports for surveillance of disease at a population health or individual level | **Disease surveillance** | Information Extraction | Thorax [1]  Mixed [2]  Cerebrovascular [3] |
|  |  | Classification | Thorax [4], [5]  Abdomen [6]  Mixed [7] |
|  | **Prioritising reports** | Classification | Other [8]  Mixed [9]  Unspecified [10], [11] |
|  | **Incidental findings** | Information Extraction | Mixed [12] |
|  |  | Classification | Thorax [13], [14]  Cerebrovascular [15] |
|  | **Patient surveillance** | Information Extraction | Thorax [16]  Abdomen [17]–[21]  Mixed [22]–[27]  Breast [28]–[33]  Unspecified [34]–[36] |
|  |  | Classification | Thorax[37], [38]  Breast [39]  Mixed [40], [41] |
|  | **Follow-up** | Information Extraction | Abdomen [42]  Unspecified [43]  Mixed [44] |
|  |  | Classification | Unspecified [45] |
| **Disease information and classification (46)**  Using imaging reports to identify information that may also be aggregated according to classification systems (no specific clinical purpose specified) | **N/A** | Information Extraction (14) | Cerebrovascular [46]–[48]  Breast [49], [50]  Abdomen [51]  Thorax [52]–[56]  Mixed [57], [58]  Unspecified [59] |
|  |  | Classification (31) | Cerebrovascular [60]–[69]  Abdomen [70]  Breast [71]–[73]  Extremities [74]–[80]  Mixed [81]–[85]  Spine [86], [87]  Thorax [88]–[91] |
| **Language discovery and knowledge structure (27)**  Investigating the structure of language in imaging reports and ways in which this may be optimised to facilitate knowledge and decision support, communication and to assist in improving NLP applications | **Knowledge support for patients/public** | Lexicon/ontology discovery | Mixed [92]–[94] |
|  | **Knowledge and decision support for clinicians** | Clustering | Mixed [95] |
|  |  | Information Extraction | Breast [96]  Mixed [97]–[100]  Thorax [101]  Cerebrovascular [102]  Unspecified [103] |
|  |  | Lexicon/Ontology | Unspecified [104] |
|  | **Variability and complexity of language for NLP purposes** | Information Extraction | Thorax [105]–[107]  Mixed [108], [109]  Unspecified [110] |
|  |  | Lexicon/ontology discovery | Unspecified [111], [112]  Spine [113]  Breast [114] |
|  |  | Classification | Unspecified [115]  Thorax) [116]–[118] |
| **Quality and compliance (20)**  Using imaging reports to assess quality and safety of radiology practice, clinical practice, and efficiency of healthcare services. | **Assessing imaging practices** | Information Extraction | Abdomen [119]  Thorax [120]  Mixed [121], [122] |
|  |  | Classification | Mixed [123]–[126]  Cerebrovascular [127]–[129]  Thorax [130], [131]  Abdomen [132]  Extremities [133] |
|  | **Audit** | Information Extraction | Mixed [134]  Cerebrovascular [135] |
|  |  | Classification | Thorax [136]  Breast [137]  Extremities [138] |
| **Cohort/Epidemiology (16)**  Using imaging reports to create patient cohorts for research purposes | **Cohort** | Classification | Abdomen [139]–[141]  Cerebrovascular [142], [143]  Mixed [144]  Spine [145] |
|  |  | Information Extraction | Unspecified [146]  Cerebrovascular [147]  Spine [148] |
|  | **Epidemiology** | Information extraction | Unspecified [149]  Cerebrovascular [150]  Abdomen [151]  Thorax [152] |
|  |  | Classification | Mixed [153]  Thorax [154] |
| **Technical NLP(10)**  Papers which do not fit to a specific category, often with a primarily technical aim. | **N/A** | Information Extraction | Mixed [155]–[157]  Thorax [158]  Unspecified [159], [160] |
|  |  | Classification | Cerebrovascular [161]  Mixed [162]  Thorax [163]  Unspecified [164] |

*Table 1 Categorisation of all publications, subcategories, and technical task*

[1] M. R. Ananda-Rajah, C. Bergmeir, F. Petitjean, M. A. Slavin, K. A. Thursky, and G. I. Webb, ‘Toward Electronic Surveillance of Invasive Mold Diseases in Hematology-Oncology Patients: An Expert System Combining Natural Language Processing of Chest Computed Tomography Reports, Microbiology, and Antifungal Drug Data’, *JCO Clin Cancer Inform*, vol. 1, pp. 1–10, 2017, doi: 10.1200/CCI.17.00011.

[2] Y. Wang, S. Mehrabi, S. Sohn, E. J. Atkinson, S. Amin, and H. Liu, ‘Natural language processing of radiology reports for identification of skeletal site-specific fractures’, *BMC Medical Informatics and Decision Making*, vol. 19, no. 3, p. 73, Apr. 2019, doi: 10.1186/s12911-019-0780-5.

[3] P. Pruitt, A. Naidech, J. Van Ornam, P. Borczuk, and W. Thompson, ‘A natural language processing algorithm to extract characteristics of subdural hematoma from head CT reports’, *Emerg Radiol*, vol. 26, no. 3, pp. 301–306, Jun. 2019, doi: 10.1007/s10140-019-01673-4.

[4] D. Baggio *et al.*, ‘Closing the gap in surveillance and audit of invasive mold diseases for antifungal stewardship using machine learning’, *Journal of Clinical Medicine*, vol. 8, no. 9, p. 1390, Sep. 2019, doi: 10.3390/jcm8091390.

[5] M. Afshar *et al.*, ‘A Computable Phenotype for Acute Respiratory Distress Syndrome Using Natural Language Processing and Machine Learning’, *AMIA Annu Symp Proc*, vol. 2018, pp. 157–165, Dec. 2018.

[6] A. B. Chapman, D. L. Mowery, D. S. Swords, Wendy. W. Chapman, and B. T. Bucher, ‘Detecting Evidence of Intra-abdominal Surgical Site Infections from Radiology Reports Using Natural Language Processing’, *AMIA Annu Symp Proc*, vol. 2017, pp. 515–524, Apr. 2018.

[7] Z. Tian, S. Sun, T. Eguale, and C. M. Rochefort, ‘Automated Extraction of VTE Events From Narrative Radiology Reports in Electronic Health Records’, *Med Care*, vol. 55, no. 10, pp. e73–e80, Oct. 2017, doi: 10.1097/MLR.0000000000000346.

[8] M. Annarumma, S. J. Withey, R. J. Bakewell, E. Pesce, V. Goh, and G. Montana, ‘Automated Triaging of Adult Chest Radiographs with Deep Artificial Neural Networks’, *Radiology*, vol. 291, no. 1, pp. 196–202, Jan. 2019, doi: 10.1148/radiol.2018180921.

[9] M. Singh, A. Murthy, and S. Singh, ‘Prioritization of Free-Text Clinical Documents: A Novel Use of a Bayesian Classifier’, *JMIR Medical Informatics*, vol. 3, no. 2, p. e17, 2015, doi: 10.2196/medinform.3793.

[10] X. Meng, C. H. Ganoe, R. T. Sieberg, Y. Y. Cheung, and S. Hassanpour, ‘Assisting radiologists with reporting urgent findings to referring physicians: A machine learning approach to identify cases for prompt communication’, *Journal of Biomedical Informatics*, vol. 93, p. 103169, May 2019, doi: 10.1016/j.jbi.2019.103169.

[11] A. Ratner, B. Hancock, J. Dunnmon, R. Goldman, and C. Ré, ‘Snorkel MeTaL: Weak Supervision for Multi-Task Learning’, in *Proceedings of the Second Workshop on Data Management for End-To-End Machine Learning*, Houston, TX, USA, 2018, vol. 3, pp. 1–4, doi: 10.1145/3209889.3209898.

[12] G. Trivedi, C. Hong, E. R. Dadashzadeh, R. M. Handzel, H. Hochheiser, and S. Visweswaran, ‘Identifying incidental findings from radiology reports of trauma patients: An evaluation of automated feature representation methods’, *International Journal of Medical Informatics*, vol. 129, pp. 81–87, Sep. 2019, doi: 10.1016/j.ijmedinf.2019.05.021.

[13] F. Farjah *et al.*, ‘An Automated Method for Identifying Individuals with a Lung Nodule Can Be Feasibly Implemented Across Health Systems’, *EGEMS (Wash DC)*, vol. 4, no. 1, p. 1254, Aug. 2016, doi: 10.13063/2327-9214.1254.

[14] B. Karunakaran, D. Misra, K. Marshall, D. Mathrawala, and S. Kethireddy, ‘Closing the loop — Finding lung cancer patients using NLP’, in *2017 IEEE International Conference on Big Data (Big Data)*, Boston, MA, Dec. 2017, pp. 2452–2461, doi: 10.1109/BigData.2017.8258203.

[15] S. Fu *et al.*, ‘Natural Language Processing for the Identification of Silent Brain Infarcts From Neuroimaging Reports’, *JMIR Medical Informatics*, vol. 7, no. 2, p. e12109, 2019, doi: 10.2196/12109.

[16] E. K. Gupta, R. Thammasudjarit, and A. Thakkinstian, ‘A Hybrid Engine for Clinical Information Extraction from Radiology Reports’, in *2019 16th International Joint Conference on Computer Science and Software Engineering (JCSSE)*, Chonburi, Thailand, Jul. 2019, pp. 293–297, doi: 10.1109/JCSSE.2019.8864178.

[17] M. Sevenster, J. Bozeman, A. Cowhy, and W. Trost, ‘A natural language processing pipeline for pairing measurements uniquely across free-text CT reports’, *Journal of Biomedical Informatics*, vol. 53, pp. 36–48, Feb. 2015, doi: 10.1016/j.jbi.2014.08.015.

[18] W. Yim, S. W. Kwan, and M. Yetisgen, ‘Classifying tumor event attributes in radiology reports’, *Journal of the Association for Information Science and Technology*, vol. 68, no. 11, pp. 2662–2674, 2017, doi: 10.1002/asi.23937.

[19] V. Cotik, H. Rodríguez, and J. Vivaldi, ‘Spanish Named Entity Recognition in the Biomedical Domain’, in *Information Management and Big Data*, Lima, Peru, 2018, vol. 898, pp. 233–248, doi: 10.1007/978-3-030-11680-4_23.

[20] W. Yim, T. Denman, S. W. Kwan, and M. Yetisgen, ‘Tumor information extraction in radiology reports for hepatocellular carcinoma patients’, *AMIA Jt Summits Transl Sci Proc*, vol. 2016, pp. 455–464, Jul. 2016.

[21] W. Yim, S. W. Kwan, and M. Yetisgen, ‘Tumor reference resolution and characteristic extraction in radiology reports for liver cancer stage prediction’, *Journal of Biomedical Informatics*, vol. 64, pp. 179–191, Dec. 2016, doi: 10.1016/j.jbi.2016.10.005.

[22] Y. Peng, K. Yan, V. Sandfort, R. M. Summers, and Z. Lu, ‘A self-attention based deep learning method for lesion attribute detection from CT reports’, in *2019 IEEE International Conference on Healthcare Informatics (ICHI)*, Xi’an, China, Jun. 2019, pp. 1–5, doi: 10.1109/ICHI.2019.8904668.

[23] S. Bozkurt, E. Alkim, I. Banerjee, and D. L. Rubin, ‘Automated Detection of Measurements and Their Descriptors in Radiology Reports Using a Hybrid Natural Language Processing Algorithm’, *J Digit Imaging*, vol. 32, no. 4, pp. 544–553, Aug. 2019, doi: 10.1007/s10278-019-00237-9.

[24] A. M. Tahmasebi *et al.*, ‘Automatic Normalization of Anatomical Phrases in Radiology Reports Using Unsupervised Learning’, *J Digit Imaging*, vol. 32, no. 1, pp. 6–18, Feb. 2019, doi: 10.1007/s10278-018-0116-5.

[25] R. C. Khor *et al.*, ‘Extracting tumour prognostic factors from a diverse electronic record dataset in genito-urinary oncology’, *International Journal of Medical Informatics*, vol. 121, pp. 53–57, Jan. 2019, doi: 10.1016/j.ijmedinf.2018.10.008.

[26] H. Oberkampf *et al.*, ‘Semantic representation of reported measurements in radiology’, *BMC Medical Informatics and Decision Making*, vol. 16, no. 1, p. 5, Jan. 2016, doi: 10.1186/s12911-016-0248-9.

[27] R. Wadia *et al.*, ‘Comparison of Natural Language Processing and Manual Coding for the Identification of Cross-Sectional Imaging Reports Suspicious for Lung Cancer’, *JCO Clin Cancer Inform*, vol. 2, Feb. 2018, doi: 10.1200/CCI.17.00069.

[28] S. Hassanpour, G. Bay, and C. P. Langlotz, ‘Characterization of Change and Significance for Clinical Findings in Radiology Reports Through Natural Language Processing’, *J Digit Imaging*, vol. 30, no. 3, pp. 314–322, Jun. 2017, doi: 10.1007/s10278-016-9931-8.

[29] R. Lacson, M. E. Goodrich, K. Harris, P. Brawarsky, and J. S. Haas, ‘Assessing Inaccuracies in Automated Information Extraction of Breast Imaging Findings’, *J Digit Imaging*, vol. 30, no. 2, pp. 228–233, Apr. 2017, doi: 10.1007/s10278-016-9927-4.

[30] Y. Liu, L.-N. Zhu, Q. Liu, C. Han, X.-D. Zhang, and X.-Y. Wang, ‘Automatic extraction of imaging observation and assessment categories from breast magnetic resonance imaging reports with natural language processing’, *Chin Med J (Engl)*, vol. 132, no. 14, pp. 1673–1680, Jul. 2019, doi: 10.1097/CM9.0000000000000301.

[31] A. Gupta, I. Banerjee, and D. L. Rubin, ‘Automatic information extraction from unstructured mammography reports using distributed semantics’, *Journal of Biomedical Informatics*, vol. 78, pp. 78–86, Feb. 2018, doi: 10.1016/j.jbi.2017.12.016.

[32] R. G. Short, J. Bralich, D. Bogaty, and N. T. Befera, ‘Comprehensive Word-Level Classification of Screening Mammography Reports Using a Neural Network Sequence Labeling Approach’, *J Digit Imaging*, vol. 32, no. 5, pp. 685–692, Oct. 2019, doi: 10.1007/s10278-018-0141-4.

[33] R. Lacson *et al.*, ‘Evaluation of an Automated Information Extraction Tool for Imaging Data Elements to Populate a Breast Cancer Screening Registry’, *J Digit Imaging*, vol. 28, no. 5, pp. 567–575, Oct. 2015, doi: 10.1007/s10278-014-9762-4.

[34] H. Zhu, I. Ch. Paschalidis, C. Hall, and A. Tahmasebi, ‘Context-Driven Concept Annotation in Radiology Reports: Anatomical Phrase Labeling’, *AMIA Jt Summits Transl Sci Proc*, vol. 2019, pp. 232–241, May 2019.

[35] M. Sevenster, J. Buurman, P. Liu, J. F. Peters, and P. J. Chang, ‘Natural Language Processing Techniques for Extracting and Categorizing Finding Measurements in Narrative Radiology Reports’, *Appl Clin Inform*, vol. 06, no. 3, pp. 600–610, 2015, doi: 10.4338/ACI-2014-11-RA-0110.

[36] H. Yang, L. Li, R. Yang, and Y. Zhou, ‘Towards Automated Knowledge Discovery of Hepatocellular Carcinoma: Extract Patient Information from Chinese Clinical Reports’, in *Proceedings of the 2nd International Conference on Medical and Health Informatics*, New York, NY, USA, Jun. 2018, pp. 111–116, doi: 10.1145/3239438.3239445.

[37] K. L. Kehl *et al.*, ‘Assessment of Deep Natural Language Processing in Ascertaining Oncologic Outcomes From Radiology Reports’, *JAMA Oncology*, vol. 5, no. 10, pp. 1421–1429, Oct. 2019, doi: 10.1001/jamaoncol.2019.1800.

[38] A. E. Gerevini *et al.*, ‘Automatic classification of radiological reports for clinical care’, *Artificial Intelligence in Medicine*, vol. 91, pp. 72–81, Sep. 2018, doi: 10.1016/j.artmed.2018.05.006.

[39] S. M. Castro *et al.*, ‘Automated annotation and classification of BI-RADS assessment from radiology reports’, *Journal of Biomedical Informatics*, vol. 69, pp. 177–187, May 2017, doi: 10.1016/j.jbi.2017.04.011.

[40] D. Martinez, M. R. Ananda-Rajah, H. Suominen, M. A. Slavin, K. A. Thursky, and L. Cavedon, ‘Automatic detection of patients with invasive fungal disease from free-text computed tomography (CT) scans’, *Journal of Biomedical Informatics*, vol. 53, pp. 251–260, Feb. 2015, doi: 10.1016/j.jbi.2014.11.009.

[41] P.-H. Chen, H. Zafar, M. Galperin-Aizenberg, and T. Cook, ‘Integrating Natural Language Processing and Machine Learning Algorithms to Categorize Oncologic Response in Radiology Reports’, *J Digit Imaging*, vol. 31, no. 2, pp. 178–184, Apr. 2018, doi: 10.1007/s10278-017-0027-x.

[42] R. Lou, D. Lalevic, C. Chambers, H. M. Zafar, and T. S. Cook, ‘Automated Detection of Radiology Reports that Require Follow-up Imaging Using Natural Language Processing Feature Engineering and Machine Learning Classification’, *J Digit Imaging*, vol. 33, no. 1, pp. 131–136, Feb. 2020, doi: 10.1007/s10278-019-00271-7.

[43] W. Lau, T. H. Payne, O. Uzuner, and M. Yetisgen, ‘Extraction and Analysis of Clinically Important Follow-up Recommendations in a Large Radiology Dataset’, *arXiv [cs.CL]*, 2019, Accessed: Oct. 30, 2020. [Online]. Available: http://arxiv.org/abs/1905.05877.

[44] L. R. Cochon *et al.*, ‘Variation in Follow-up Imaging Recommendations in Radiology Reports: Patient, Modality, and Radiologist Predictors’, *Radiology*, vol. 291, no. 3, pp. 700–707, May 2019, doi: 10.1148/radiol.2019182826.

[45] E. Carrodeguas, R. Lacson, W. Swanson, and R. Khorasani, ‘Use of Machine Learning to Identify Follow-Up Recommendations in Radiology Reports’, *J Am Coll Radiol*, vol. 16, no. 3, pp. 336–343, Mar. 2019, doi: 10.1016/j.jacr.2018.10.020.

[46] I. Banerjee, S. Madhavan, R. E. Goldman, and D. L. Rubin, ‘Intelligent Word Embeddings of Free-Text Radiology Reports’, *AMIA Annu Symp Proc*, pp. 411–420, 2017.

[47] P. J. Gorinski *et al.*, ‘Named Entity Recognition for Electronic Health Records: A Comparison of Rule-based and Machine Learning Approaches’, *arXiv:1903.03985 [cs.CL]*, Jun. 2019, Accessed: Oct. 30, 2020. [Online]. Available: http://arxiv.org/abs/1903.03985.

[48] B. Alex, C. Grover, R. Tobin, C. Sudlow, G. Mair, and W. Whiteley, ‘Text mining brain imaging reports’, *Journal of Biomedical Semantics*, vol. 10, no. 1, p. 23, Nov. 2019, doi: 10.1186/s13326-019-0211-7.

[49] S. Miao *et al.*, ‘Extraction of BI-RADS findings from breast ultrasound reports in Chinese using deep learning approaches’, *International Journal of Medical Informatics*, vol. 119, pp. 17–21, Nov. 2018, doi: 10.1016/j.ijmedinf.2018.08.009.

[50] I. Banerjee, S. Bozkurt, J. L. Caswell-Jin, A. W. Kurian, and D. L. Rubin, ‘Natural Language Processing Approaches to Detect the Timeline of Metastatic Recurrence of Breast Cancer’, *JCO Clinical Cancer Informatics*, no. 3, pp. 1–12, Oct. 2019, doi: 10.1200/CCI.19.00034.

[51] L. Chen, L. Song, Y. Shao, D. Li, and K. Ding, ‘Using natural language processing to extract clinically useful information from Chinese electronic medical records’, *International Journal of Medical Informatics*, vol. 124, pp. 6–12, Apr. 2019, doi: 10.1016/j.ijmedinf.2019.01.004.

[52] R. Rafeh and M. Ahmadi, ‘A New Approach for Classifying Radiology Reports’, *Journal of Medical Imaging and Health Informatics*, vol. 5, no. 2, pp. 257–263, Apr. 2015, doi: 10.1166/jmihi.2015.1383.

[53] V. Liventsev, I. Fedulova, and D. Dylov, ‘Deep Text Prior: Weakly Supervised Learning for Assertion Classification’, in *Artificial Neural Networks and Machine Learning – ICANN 2019: Workshop and Special Sessions*, Munich, Germany, 2019, pp. 243–257, doi: 10.1007/978-3-030-30493-5_26.

[54] S. Meystre, R. Gouripeddi, J. Tieder, J. Simmons, R. Srivastava, and S. Shah, ‘Enhancing Comparative Effectiveness Research With Automated Pediatric Pneumonia Detection in a Multi-Institutional Clinical Repository: A PHIS+ Pilot Study’, *Journal of Medical Internet Research*, vol. 19, no. 5, p. e162, 2017, doi: 10.2196/jmir.6887.

[55] Y. Kim *et al.*, ‘Extraction of left ventricular ejection fraction information from various types of clinical reports’, *Journal of Biomedical Informatics*, vol. 67, pp. 42–48, Mar. 2017, doi: 10.1016/j.jbi.2017.01.017.

[56] O. V. Patterson, M. S. Freiberg, M. Skanderson, S. J. Fodeh, C. A. Brandt, and S. L. DuVall, ‘Unlocking echocardiogram measurements for heart disease research through natural language processing’, *BMC Cardiovascular Disorders*, vol. 17, no. 1, p. 151, Jun. 2017, doi: 10.1186/s12872-017-0580-8.

[57] R. B. Dantes *et al.*, ‘Improved Identification of Venous Thromboembolism From Electronic Medical Records Using a Novel Information Extraction Software Platform’, *Medical Care*, vol. 56, no. 9, p. e54, Sep. 2018, doi: 10.1097/MLR.0000000000000831.

[58] R. R. de Haan *et al.*, ‘Patient-specific workup of adrenal incidentalomas’, *European Journal of Radiology Open*, vol. 4, pp. 108–114, Jan. 2017, doi: 10.1016/j.ejro.2017.08.002.

[59] N. Collier, A. Oellrich, and T. Groza, ‘Concept selection for phenotypes and diseases using learn to rank’, *Journal of Biomedical Semantics*, vol. 6, no. 1, p. 24, Jun. 2015, doi: 10.1186/s13326-015-0019-z.

[60] K. Jnawali, M. R. Arbabshirani, A. E. Ulloa, N. Rao, and A. A. Patel, ‘Automatic Classification of Radiological Report for Intracranial Hemorrhage’, in *2019 IEEE 13th International Conference on Semantic Computing (ICSC)*, Newport Beach, CA, USA, Jan. 2019, pp. 187–190, doi: 10.1109/ICOSC.2019.8665578.

[61] N. Deshmukh *et al.*, ‘Semi-Supervised Natural Language Approach for Fine-Grained Classification of Medical Reports’, *arXiv:1910.13573 [cs.LG]*, Nov. 2019, Accessed: Oct. 30, 2020. [Online]. Available: http://arxiv.org/abs/1910.13573.

[62] C. Kim, V. Zhu, J. Obeid, and L. Lenert, ‘Natural language processing and machine learning algorithm to identify brain MRI reports with acute ischemic stroke’, *PLOS ONE*, vol. 14, no. 2, p. e0212778, Feb. 2019, doi: 10.1371/journal.pone.0212778.

[63] R. Garg, E. Oh, A. Naidech, K. Kording, and S. Prabhakaran, ‘Automating Ischemic Stroke Subtype Classification Using Machine Learning and Natural Language Processing’, *Journal of Stroke and Cerebrovascular Diseases*, vol. 28, no. 7, pp. 2045–2051, Jul. 2019, doi: 10.1016/j.jstrokecerebrovasdis.2019.02.004.

[64] B. Shin, F. H. Chokshi, T. Lee, and J. D. Choi, ‘Classification of radiology reports using neural attention models’, in *2017 International Joint Conference on Neural Networks (IJCNN)*, Anchorage, AK, May 2017, pp. 4363–4370, doi: 10.1109/IJCNN.2017.7966408.

[65] E. Wheater, G. Mair, C. Sudlow, B. Alex, C. Grover, and W. Whiteley, ‘A validated natural language processing algorithm for brain imaging phenotypes from radiology reports in UK electronic health records’, *BMC Med Inform Decis Mak*, vol. 19, no. 1, p. 184, Sep. 2019, doi: 10.1186/s12911-019-0908-7.

[66] J. T. Senders *et al.*, ‘Natural Language Processing for Automated Quantification of Brain Metastases Reported in Free-Text Radiology Reports’, *JCO Clinical Cancer Informatics*, no. 3, pp. 1–9, Apr. 2019, doi: 10.1200/CCI.18.00138.

[67] J. Zech *et al.*, ‘Natural Language–based Machine Learning Models for the Annotation of Clinical Radiology Reports’, *Radiology*, vol. 287, no. 2, pp. 570–580, Jan. 2018, doi: 10.1148/radiol.2018171093.

[68] E. S. Kayi, K. Yadav, J. M. Chamberlain, and H.-A. Choi, ‘Topic Modeling for Classification of Clinical Reports’, *arXiv:1706.06177 [cs.CL]*, Jun. 2017, Accessed: Oct. 30, 2020. [Online]. Available: http://arxiv.org/abs/1706.06177.

[69] M. Kłos, J. Żyłkowski, and D. Spinczyk, ‘Automatic Classification of Text Documents Presenting Radiology Examinations’, in *Proceedings 6th International Conference Information Technology in Biomedicine(ITIB’2018)*, Kamień Śląski, Poland, 2018, pp. 495–505, doi: 10.1007/978-3-319-91211-0_43.

[70] W. Yim, S. W. Kwan, G. Johnson, and M. Yetisgen, ‘Classification of hepatocellular carcinoma stages from free-text clinical and radiology reports’, *AMIA Annu Symp Proc*, vol. 2017, pp. 1858–1867, Apr. 2018.

[71] I. Banerjee, S. Bozkurt, E. Alkim, H. Sagreiya, A. W. Kurian, and D. L. Rubin, ‘Automatic inference of BI-RADS final assessment categories from narrative mammography report findings’, *Journal of Biomedical Informatics*, vol. 92, p. 103137, Apr. 2019, doi: 10.1016/j.jbi.2019.103137.

[72] T. A. Patel *et al.*, ‘Correlating mammographic and pathologic findings in clinical decision support using natural language processing and data mining methods’, *Cancer*, vol. 123, no. 1, pp. 114–121, Jan. 2017, doi: 10.1002/cncr.30245.

[73] S. Bozkurt, F. Gimenez, E. S. Burnside, K. H. Gulkesen, and D. L. Rubin, ‘Using automatically extracted information from mammography reports for decision-support’, *Journal of Biomedical Informatics*, vol. 62, pp. 224–231, Aug. 2016, doi: 10.1016/j.jbi.2016.07.001.

[74] R. W. Grundmeier *et al.*, ‘Identification of Long Bone Fractures in Radiology Reports Using Natural Language Processing to support Healthcare Quality Improvement’, *Appl Clin Inform*, vol. 07, no. 4, pp. 1051–1068, 2016, doi: 10.4338/ACI-2016-08-RA-0129.

[75] C. Lee, Y. Kim, Y. S. Kim, and J. Jang, ‘Automatic Disease Annotation From Radiology Reports Using Artificial Intelligence Implemented by a Recurrent Neural Network’, *American Journal of Roentgenology*, vol. 212, no. 4, pp. 734–740, Jan. 2019, doi: 10.2214/AJR.18.19869.

[76] H. Hassanzadeh, M. Kholghi, A. Nguyen, and K. Chu, ‘Clinical Document Classification Using Labeled and Unlabeled Data Across Hospitals’, *AMIA Annu Symp Proc*, vol. 2018, pp. 545–554, Dec. 2018.

[77] J. Swartz, C. Koziatek, J. Theobald, S. Smith, and E. Iturrate, ‘Creation of a simple natural language processing tool to support an imaging utilization quality dashboard’, *International Journal of Medical Informatics*, vol. 101, pp. 93–99, May 2017, doi: 10.1016/j.ijmedinf.2017.02.011.

[78] J. Fiebeck, H. Laser, H. B. Winther, and S. Gerbel, ‘Leaving No Stone Unturned: Using Machine Learning Based Approaches for Information Extraction from Full Texts of a Research Data Warehouse’, in *13th International Conference Data Integration in the Life Sciences (DILS 2018)*, Hannover, Germany, 2018, pp. 50–58, doi: 10.1007/978-3-030-06016-9_5.

[79] J. A. Gálvez *et al.*, ‘The use of natural language processing on pediatric diagnostic radiology reports in the electronic health record to identify deep venous thrombosis in children’, *J Thromb Thrombolysis*, vol. 44, no. 3, pp. 281–290, Oct. 2017, doi: 10.1007/s11239-017-1532-y.

[80] H. Hassanzadeh, A. Nguyen, S. Karimi, and K. Chu, ‘Transferability of artificial neural networks for clinical document classification across hospitals: A case study on abnormality detection from radiology reports’, *Journal of Biomedical Informatics*, vol. 85, pp. 68–79, Sep. 2018, doi: 10.1016/j.jbi.2018.07.017.

[81] R. Kavuluru, A. Rios, and Y. Lu, ‘An empirical evaluation of supervised learning approaches in assigning diagnosis codes to electronic medical records’, *Artificial Intelligence in Medicine*, vol. 65, no. 2, pp. 155–166, Oct. 2015, doi: 10.1016/j.artmed.2015.04.007.

[82] S. Karimi, X. Dai, H. Hassanzadeh, and A. Nguyen, ‘Automatic Diagnosis Coding of Radiology Reports: A Comparison of Deep Learning and Conventional Classification Methods’, in *BioNLP 2017*, Vancouver, Canada, Aug. 2017, pp. 328–332, doi: 10.18653/v1/W17-2342.

[83] S. Kocbek *et al.*, ‘Evaluating classification power of linked admission data sources with text mining’, in *Proceedings of the Scientific Stream at Big Data in Health Analytics 2015 (BigData 2015)*, Swissotel Sydney, Sydney, Australia, 2015, vol. 1468, pp. 1–7, [Online]. Available: http://ceur-ws.org/Vol-1468/.

[84] G. S. Krishnan and S. Kamath S., ‘Ontology-driven Text Feature Modeling for Disease Prediction using Unstructured Radiological Notes’, *Computación y Sistemas*, vol. 23, no. 3, Art. no. 3, Sep. 2019, doi: 10.13053/cys-23-3-3238.

[85] S. Kocbek *et al.*, ‘Text mining electronic hospital records to automatically classify admissions against disease: Measuring the impact of linking data sources’, *Journal of Biomedical Informatics*, vol. 64, pp. 158–167, Dec. 2016, doi: 10.1016/j.jbi.2016.10.008.

[86] W. K. Tan *et al.*, ‘Comparison of Natural Language Processing Rules-based and Machine-learning Systems to Identify Lumbar Spine Imaging Findings Related to Low Back Pain’, *Academic Radiology*, vol. 25, no. 11, pp. 1422–1432, Nov. 2018, doi: 10.1016/j.acra.2018.03.008.

[87] J. Pandya, K. Ganda, L. Ridley, and M. J. Seibel, ‘Identification of Patients with Osteoporotic Vertebral Fractures via Simple Text Search of Routine Radiology Reports’, *Calcif Tissue Int*, vol. 105, no. 2, pp. 156–160, Aug. 2019, doi: 10.1007/s00223-019-00557-6.

[88] S. E. Beyer *et al.*, ‘Automatic Lung-RADS^TM^ classification with a natural language processing system’, *J Thorac Dis*, vol. 9, no. 9, pp. 3114–3122, Sep. 2017, doi: 10.21037/jtd.2017.08.13.

[89] I. Banerjee *et al.*, ‘Comparative effectiveness of convolutional neural network (CNN) and recurrent neural network (RNN) architectures for radiology text report classification’, *Artificial Intelligence in Medicine*, vol. 97, pp. 79–88, Jun. 2019, doi: 10.1016/j.artmed.2018.11.004.

[90] M. C. Chen *et al.*, ‘Deep Learning to Classify Radiology Free-Text Reports’, *Radiology*, vol. 286, no. 3, pp. 845–852, Nov. 2017, doi: 10.1148/radiol.2017171115.

[91] R. M. Dunne *et al.*, ‘Effect of Evidence-based Clinical Decision Support on the Use and Yield of CT Pulmonary Angiographic Imaging in Hospitalized Patients’, *Radiology*, vol. 276, no. 1, pp. 167–174, Feb. 2015, doi: 10.1148/radiol.15141208.

[92] T. Martin-Carreras and C. E. Kahn, ‘Coverage and Readability of Information Resources to Help Patients Understand Radiology Reports’, *Journal of the American College of Radiology*, vol. 15, no. 12, pp. 1681–1686, Dec. 2018, doi: 10.1016/j.jacr.2017.11.019.

[93] B. Qenam, T. Y. Kim, M. J. Carroll, and M. Hogarth, ‘Text Simplification Using Consumer Health Vocabulary to Generate Patient-Centered Radiology Reporting: Translation and Evaluation’, *Journal of Medical Internet Research*, vol. 19, no. 12, p. e417, 2017, doi: 10.2196/jmir.8536.

[94] M. Lafourcade and L. Ramadier, ‘Radiological text simplification using a general knowledge base’, presented at the 18th International Conference on Computational Linguistics and Intelligent Text Processing (CICLing 2017), 2017, doi: https://doi.org/10.1007/978-3-319-77116-8_46.

[95] S. Hassanpour and C. P. Langlotz, ‘Unsupervised Topic Modeling in a Large Free Text Radiology Report Repository’, *J Digit Imaging*, vol. 29, no. 1, pp. 59–62, Feb. 2016, doi: 10.1007/s10278-015-9823-3.

[96] A. Comelli, L. Agnello, and S. Vitabile, ‘An ontology-based retrieval system for mammographic reports’, in *2015 IEEE Symposium on Computers and Communication (ISCC)*, Larnaca, Jul. 2015, pp. 1001–1006, doi: 10.1109/ISCC.2015.7405644.

[97] M. D. Kovacs, J. Mesterhazy, D. Avrin, T. Urbania, and J. Mongan, ‘Correlate: A PACS- and EHR-integrated Tool Leveraging Natural Language Processing to Provide Automated Clinical Follow-up’, *RadioGraphics*, vol. 37, no. 5, pp. 1451–1460, Sep. 2017, doi: 10.1148/rg.2017160195.

[98] S. Lalithsena *et al.*, ‘Feedback-Driven Radiology Exam Report Retrieval with Semantics’, in *Proceedings of the 2015 International Conference on Healthcare Informatics*, Dallas, Texas, 2015, pp. 233–242, doi: 10.1109/ICHI.2015.35.

[99] Y. Zhao, N. J. Fesharaki, H. Liu, and J. Luo, ‘Using data-driven sublanguage pattern mining to induce knowledge models: application in medical image reports knowledge representation’, *BMC Medical Informatics and Decision Making*, vol. 18, no. 1, p. 61, Jul. 2018, doi: 10.1186/s12911-018-0645-3.

[100] L. Shi, T. Ling, and J. Zhang, ‘Semantic information extracting system for classification of radiological reports in radiology information system (RIS)’, in *Medical Imaging 2016: PACS and Imaging Informatics: Next Generation and Innovations*, San Diego, California, United States, Mar. 2016, vol. 9789, pp. 162–175, doi: 10.1117/12.2216183.

[101] Y. Hong and J. Zhang, ‘Investigation of Terminology Coverage in Radiology Reporting Templates and Free‐text Reports’, *International Journal of Knowledge Content Development & Technology*, vol. 5, pp. 5–14, 2015, doi: 10.5865/IJKCT.2015.5.1.005.

[102] W. Scuba *et al.*, ‘Knowledge Author: facilitating user-driven, domain content development to support clinical information extraction’, *Journal of Biomedical Semantics*, vol. 7, no. 1, p. 42, Jun. 2016, doi: 10.1186/s13326-016-0086-9.

[103] E. Monteiro, P. Sernadela, S. Matos, C. Costa, and J. L. Oliveira, ‘Semantic Knowledge Base Construction from Radiology Reports’, in *Proceedings of the 9th International Joint Conference on Biomedical Engineering Systems and Technologies HEALTHINF, (BIOSTEC 2016)*, Rome,Italy, 2016, vol. 5, pp. 345–352, [Online]. Available: https://www.scitepress.org/Link.aspx?doi=10.5220/0005709503450352.

[104] J. Hostetter, K. Wang, E. Siegel, J. Durack, and J. J. Morrison, ‘Using Standardized Lexicons for Report Template Validation with LexMap, a Web-based Application’, *J Digit Imaging*, vol. 28, no. 3, pp. 309–314, Jun. 2015, doi: 10.1007/s10278-014-9760-6.

[105] R. G. Short, N. T. Befera, J. K. Hoang, and T. D. Tailor, ‘A Normal Thyroid by Any Other Name: Linguistic Analysis of Statements Describing a Normal Thyroid Gland from Noncontrast Chest CT Reports’, *Journal of the American College of Radiology*, vol. 15, no. 11, pp. 1642–1647, Nov. 2018, doi: 10.1016/j.jacr.2018.04.016.

[106] E. Johnson, W. C. Baughman, and G. Ozsoyoglu, ‘A method for imputation of semantic class in diagnostic radiology text’, in *2015 IEEE International Conference on Bioinformatics and Biomedicine (BIBM)*, Washington, DC, Nov. 2015, pp. 750–755, doi: 10.1109/BIBM.2015.7359780.

[107] Z. Xie *et al.*, ‘Introducing Information Extraction to Radiology Information Systems to Improve the Efficiency on Reading Reports’, *Methods Inf Med*, vol. 58, no. 2–03, pp. 94–106, 2019, doi: 10.1055/s-0039-1694992.

[108] L. F. Donnelly, R. Grzeszczuk, C. V. Guimaraes, W. Zhang, and G. S. Bisset III, ‘Using a Natural Language Processing and Machine Learning Algorithm Program to Analyze Inter-Radiologist Report Style Variation and Compare Variation Between Radiologists When Using Highly Structured Versus More Free Text Reporting’, *Current Problems in Diagnostic Radiology*, vol. 48, no. 6, pp. 524–530, Nov. 2019, doi: 10.1067/j.cpradiol.2018.09.005.

[109] S. Mujjiga, V. Krishna, K. Chakravarthi, and V. J, ‘Identifying Semantics in Clinical Reports Using Neural Machine Translation’, *Proceedings of the AAAI Conference on Artificial Intelligence*, vol. 33, no. 01, Art. no. 01, Jul. 2019, doi: 10.1609/aaai.v33i01.33019552.

[110] M. Lafourcade and L. Ramadier, ‘Semantic RelationExtraction with Semantic Patterns: Experiment on Radiology Report’, Portorož, Slovenia, 2016, [Online]. Available: https://hal.archives-ouvertes.fr/hal-01382320.

[111] B. Percha, Y. Zhang, S. Bozkurt, D. Rubin, R. B. Altman, and C. P. Langlotz, ‘Expanding a radiology lexicon using contextual patterns in radiology reports’, *J Am Med Inform Assoc*, vol. 25, no. 6, pp. 679–685, Jun. 2018, doi: 10.1093/jamia/ocx152.

[112] Y. Pershad *et al.*, ‘Using Naïve Bayesian Analysis to Determine Imaging Characteristics of KRAS Mutations in Metastatic Colon Cancer’, *Diagnostics (Basel)*, vol. 7, no. 3, p. 50, Sep. 2017, doi: 10.3390/diagnostics7030050.

[113] F. Barbosa, A. J. Traina, and V. F. Muglia, ‘Meta-generalis: A novel method for structuring information from radiology reports’, *Appl Clin Inform*, vol. 07, no. 3, pp. 803–816, 2016, doi: 10.4338/ACI-2016-03-RA-0037.

[114] H. Bulu, D. A. Sippo, J. M. Lee, E. S. Burnside, and D. L. Rubin, ‘Proposing New RadLex Terms by Analyzing Free-Text Mammography Reports’, *J Digit Imaging*, vol. 31, no. 5, pp. 596–603, Oct. 2018, doi: 10.1007/s10278-018-0064-0.

[115] Cotik Viviana, D. Filippo, and J. Castano, ‘An Approach for Automatic Classification of Radiology Reports in Spanish.’, *Stud Health Technol Inform*, vol. 216, pp. 634–638, Jan. 2015.

[116] A. Spandorfer *et al.*, ‘Deep learning to convert unstructured CT pulmonary angiography reports into structured reports’, *Eur Radiol Exp*, vol. 3, no. 1, p. 37, Sep. 2019, doi: 10.1186/s41747-019-0118-1.

[117] M. D. Huesch, R. Cherian, S. Labib, and R. Mahraj, ‘Evaluating Report Text Variation and Informativeness: Natural Language Processing of CT Chest Imaging for Pulmonary Embolism’, *Journal of the American College of Radiology*, vol. 15, no. 3, Part B, pp. 554–562, Mar. 2018, doi: 10.1016/j.jacr.2017.12.017.

[118] I. Banerjee, M. C. Chen, M. P. Lungren, and D. L. Rubin, ‘Radiology report annotation using intelligent word embeddings: Applied to multi-institutional chest CT cohort’, *Journal of Biomedical Informatics*, vol. 77, pp. 11–20, Jan. 2018, doi: 10.1016/j.jbi.2017.11.012.

[119] M. D. Bobbin, I. K. Ip, V. A. Sahni, A. B. Shinagare, and R. Khorasani, ‘Focal Cystic Pancreatic Lesion Follow-up Recommendations After Publication of ACR White Paper on Managing Incidental Findings’, *Journal of the American College of Radiology*, vol. 14, no. 6, pp. 757–764, Jun. 2017, doi: 10.1016/j.jacr.2017.01.044.

[120] J. L. Kwan, D. Yermak, L. Markell, N. S. Paul, K. J. Shojania, and P. Cram, ‘Follow Up of Incidental High-Risk Pulmonary Nodules on Computed Tomography Pulmonary Angiography at Care Transitions.’, *Journal of Hospital Medicine*, vol. 14, no. 6, pp. 349–352, Jun. 2019, doi: 10.12788/jhm.3128.

[121] T. Mabotuwana, C. S. Hall, J. Tieder, and M. L. Gunn, ‘Improving Quality of Follow-Up Imaging Recommendations in Radiology’, *AMIA Annu Symp Proc*, vol. 2017, pp. 1196–1204, Apr. 2018.

[122] T. Mabotuwana, V. Hombal, S. Dalal, C. S. Hall, and M. Gunn, ‘Determining Adherence to Follow-up Imaging Recommendations’, *Journal of the American College of Radiology*, vol. 15, no. 3, Part A, pp. 422–428, Mar. 2018, doi: 10.1016/j.jacr.2017.11.022.

[123] S. C. Shelmerdine, M. Singh, W. Norman, R. Jones, N. J. Sebire, and O. J. Arthurs, ‘Automated data extraction and report analysis in computer-aided radiology audit: practice implications from post-mortem paediatric imaging’, *Clinical Radiology*, vol. 74, no. 9, p. 733.e11-733.e18, Sep. 2019, doi: 10.1016/j.crad.2019.04.021.

[124] S. Dalal *et al.*, ‘Determining Follow-Up Imaging Study Using Radiology Reports’, *J Digit Imaging*, vol. 33, no. 1, pp. 121–130, Feb. 2020, doi: 10.1007/s10278-019-00260-w.

[125] H. Trivedi, J. Mesterhazy, B. Laguna, T. Vu, and J. H. Sohn, ‘Automatic Determination of the Need for Intravenous Contrast in Musculoskeletal MRI Examinations Using IBM Watson’s Natural Language Processing Algorithm’, *J Digit Imaging*, vol. 31, no. 2, pp. 245–251, Apr. 2018, doi: 10.1007/s10278-017-0021-3.

[126] S. Hassanpour and C. P. Langlotz, ‘Predicting High Imaging Utilization Based on Initial Radiology Reports:: A Feasibility Study of Machine Learning’, *Academic Radiology*, vol. 23, no. 1, pp. 84–89, Jan. 2016, doi: 10.1016/j.acra.2015.09.014.

[127] A. D. Brown and T. R. Marotta, ‘A Natural Language Processing-based Model to Automate MRI Brain Protocol Selection and Prioritization’, *Academic Radiology*, vol. 24, no. 2, pp. 160–166, Feb. 2017, doi: 10.1016/j.acra.2016.09.013.

[128] A. D. Brown and T. R. Marotta, ‘Using machine learning for sequence-level automated MRI protocol selection in neuroradiology’, *J Am Med Inform Assoc*, vol. 25, no. 5, pp. 568–571, May 2018, doi: 10.1093/jamia/ocx125.

[129] A. Y. Zhang, S. S. W. Lam, N. Liu, Y. Pang, L. L. Chan, and P. H. Tang, ‘Development of a Radiology Decision Support System for the Classification of MRI Brain Scans’, in *2018 IEEE/ACM 5th International Conference on Big Data Computing Applications and Technologies (BDCAT)*, Dec. 2018, pp. 107–115, doi: 10.1109/BDCAT.2018.00021.

[130] S. K. Kang *et al.*, ‘Natural Language Processing for Identification of Incidental Pulmonary Nodules in Radiology Reports’, *Journal of the American College of Radiology*, vol. 16, no. 11, pp. 1587–1594, Nov. 2019, doi: 10.1016/j.jacr.2019.04.026.

[131] Z. Yan, I. K. Ip, A. S. Raja, A. Gupta, J. M. Kosowsky, and R. Khorasani, ‘Yield of CT Pulmonary Angiography in the Emergency Department When Providers Override Evidence-based Clinical Decision Support’, *Radiology*, vol. 282, no. 3, pp. 717–725, Sep. 2016, doi: 10.1148/radiol.2016151985.

[132] A. D. Brown and J. R. Kachura, ‘Natural Language Processing of Radiology Reports in Patients With Hepatocellular Carcinoma to Predict Radiology Resource Utilization’, *Journal of the American College of Radiology*, vol. 16, no. 6, pp. 840–844, Jun. 2019, doi: 10.1016/j.jacr.2018.12.004.

[133] S. Hassanpour, C. P. Langlotz, T. J. Amrhein, N. T. Befera, and M. P. Lungren, ‘Performance of a Machine Learning Classifier of Knee MRI Reports in Two Large Academic Radiology Practices: A Tool to Estimate Diagnostic Yield’, *American Journal of Roentgenology*, vol. 208, no. 4, pp. 750–753, Jan. 2017, doi: 10.2214/AJR.16.16128.

[134] M. J. Minn, A. R. Zandieh, and R. W. Filice, ‘Improving Radiology Report Quality by Rapidly Notifying Radiologist of Report Errors’, *J Digit Imaging*, vol. 28, no. 4, pp. 492–498, Aug. 2015, doi: 10.1007/s10278-015-9781-9.

[135] M. E. Maros *et al.*, ‘Objective Comparison Using Guideline-based Query of Conventional Radiological Reports and Structured Reports’, *In Vivo*, vol. 32, no. 4, pp. 843–849, Jan. 2018, doi: 10.21873/invivo.11318.

[136] M. E. Heilbrun, B. E. Chapman, E. Narasimhan, N. Patel, and D. Mowery, ‘Feasibility of Natural Language Processing–Assisted Auditing of Critical Findings in Chest Radiology’, *Journal of the American College of Radiology*, vol. 16, no. 9, Part B, pp. 1299–1304, Sep. 2019, doi: 10.1016/j.jacr.2019.05.038.

[137] W. Hsu, S. X. Han, C. W. Arnold, A. A. Bui, and D. R. Enzmann, ‘A data-driven approach for quality assessment of radiologic interpretations’, *J Am Med Inform Assoc*, vol. 23, no. e1, pp. e152–e156, Apr. 2016, doi: 10.1093/jamia/ocv161.

[138] B. Koopman *et al.*, ‘Automated Reconciliation of Radiology Reports and Discharge Summaries’, *AMIA Annu Symp Proc*, vol. 2015, pp. 775–784, Nov. 2015.

[139] J. S. Redman *et al.*, ‘Accurate Identification of Fatty Liver Disease in Data Warehouse Utilizing Natural Language Processing’, *Dig Dis Sci*, vol. 62, no. 10, pp. 2713–2718, Oct. 2017, doi: 10.1007/s10620-017-4721-9.

[140] A. Y. Li and N. Elliot, ‘Natural language processing to identify ureteric stones in radiology reports’, *Journal of Medical Imaging and Radiation Oncology*, vol. 63, no. 3, pp. 307–310, 2019, doi: 10.1111/1754-9485.12861.

[141] Y. Sada, J. Hou, P. Richardson, H. El-Serag, and J. Davila, ‘Validation of Case Finding Algorithms for Hepatocellular Cancer from Administrative Data and Electronic Health Records using Natural Language Processing’, *Med Care*, vol. 54, no. 2, pp. e9–e14, Feb. 2016, doi: 10.1097/MLR.0b013e3182a30373.

[142] K. Yadav, E. Sarioglu, H.-A. Choi, W. B. Cartwright, P. S. Hinds, and J. M. Chamberlain, ‘Automated Outcome Classification of Computed Tomography Imaging Reports for Pediatric Traumatic Brain Injury’, *Academic Emergency Medicine*, vol. 23, no. 2, pp. 171–178, 2016, doi: 10.1111/acem.12859.

[143] A. J. Masino, R. W. Grundmeier, J. W. Pennington, J. A. Germiller, and E. B. Crenshaw, ‘Temporal bone radiology report classification using open source machine learning and natural langue processing libraries’, *BMC Medical Informatics and Decision Making*, vol. 16, no. 1, p. 65, Jun. 2016, doi: 10.1186/s12911-016-0306-3.

[144] J. Bates, S. J. Fodeh, C. A. Brandt, and J. A. Womack, ‘Classification of radiology reports for falls in an HIV study cohort’, *J Am Med Inform Assoc*, vol. 23, no. e1, pp. e113–e117, Apr. 2016, doi: 10.1093/jamia/ocv155.

[145] H. T. Huhdanpaa *et al.*, ‘Using Natural Language Processing of Free-Text Radiology Reports to Identify Type 1 Modic Endplate Changes’, *J Digit Imaging*, vol. 31, no. 1, pp. 84–90, Feb. 2018, doi: 10.1007/s10278-017-0013-3.

[146] V. I. Valtchinov, R. Lacson, A. Wang, and R. Khorasani, ‘Comparing Artificial Intelligence Approaches to Retrieve Clinical Reports Documenting Implantable Devices Posing MRI Safety Risks’, *Journal of the American College of Radiology*, vol. 17, no. 2, pp. 272–279, Feb. 2020, doi: 10.1016/j.jacr.2019.07.018.

[147] M. Mahan *et al.*, ‘tbiExtractor: A framework for extracting traumatic brain injury common data elements from radiology reports’, *bioRxiv 585331*, 2019, doi: 10.1101/585331.

[148] W. K. Tan and P. J. Heagerty, ‘Surrogate-guided sampling designs for classification of rare outcomes from electronic medical records data’, *arXiv:1904.00412 [stat.ME]*, Mar. 2019, Accessed: Oct. 30, 2020. [Online]. Available: http://arxiv.org/abs/1904.00412.

[149] Charles E. Khan Jr., ‘An Ontology-Based Approach to Estimate the Frequency of Rare Diseases in Narrative-Text Radiology Reports’, in *MEDINFO:2017 Precision Healthcare through Informatics*, vol. 245, IOS Press Ebooks, pp. 896–900.

[150] N. Noorbakhsh-Sabet *et al.*, ‘Racial Difference in Cerebral Microbleed Burden Among a Patient Population in the Mid-South United States’, *Journal of Stroke and Cerebrovascular Diseases*, vol. 27, no. 10, pp. 2657–2661, Oct. 2018, doi: 10.1016/j.jstrokecerebrovasdis.2018.05.031.

[151] I. Goldshtein, G. Chodick, I. Kochba, N. Gal, M. Webb, and O. Shibolet, ‘Identification and Characterization of Nonalcoholic Fatty Liver Disease’, *Clinical Gastroenterology and Hepatology*, vol. 18, no. 8, pp. 1887–1889, Jul. 2020, doi: 10.1016/j.cgh.2019.08.007.

[152] R. M. Van Haren *et al.*, ‘Ground Glass Lesions on Chest Imaging: Evaluation of Reported Incidence in Cancer Patients Using Natural Language Processing’, *The Annals of Thoracic Surgery*, vol. 107, no. 3, pp. 936–940, Mar. 2019, doi: 10.1016/j.athoracsur.2018.09.016.

[153] K. Brizzi *et al.*, ‘Natural Language Processing to Assess Palliative Care and End-of-Life Process Measures in Patients With Breast Cancer With Leptomeningeal Disease’, *American Journal of Hospice and Palliative Medicine*, vol. 37, no. 5, pp. 371–376, 2019, doi: https://doi.org/10.1177/1049909119885585.

[154] M. K. Gould *et al.*, ‘Recent Trends in the Identification of Incidental Pulmonary Nodules’, *Am J Respir Crit Care Med*, vol. 192, no. 10, pp. 1208–1214, Jul. 2015, doi: 10.1164/rccm.201505-0990OC.

[155] W. Koza *et al.*, ‘Automatic Detection of Negated Findings in Radiological Reports for Spanish Language: Methodology Based on Lexicon-Grammatical Information Processing’, *J Digit Imaging*, vol. 32, no. 1, pp. 19–29, Feb. 2019, doi: 10.1007/s10278-018-0113-8.

[156] J. Zech, J. Forde, J. J. Titano, D. Kaji, A. Costa, and E. K. Oermann, ‘Detecting insertion, substitution, and deletion errors in radiology reports using neural sequence-to-sequence models’, *Ann Transl Med*, vol. 7, no. 11, Jun. 2019, doi: 10.21037/atm.2018.08.11.

[157] J. M. Steinkamp, C. Chambers, D. Lalevic, H. M. Zafar, and T. S. Cook, ‘Toward Complete Structured Information Extraction from Radiology Reports Using Machine Learning’, *J Digit Imaging*, vol. 32, no. 4, pp. 554–564, Aug. 2019, doi: 10.1007/s10278-019-00234-y.

[158] Y. Peng, X. Wang, L. Lu, M. Bagheri, R. Summers, and Z. Lu, ‘NegBio: a high-performance tool for negation and uncertainty detection in radiology reports’, *AMIA Jt Summits Transl Sci Proc*, vol. 2017, pp. 188–196, May 2018.

[159] E. Sergeeva, H. Zhu, P. Prinsen, and A. Tahmasebi, ‘Negation Scope Detection in Clinical Notes and Scientific Abstracts: A Feature-enriched LSTM-based Approach’, *AMIA Jt Summits Transl Sci Proc*, vol. 2019, pp. 212–221, May 2019.

[160] V. Cotik, V. Stricker, J. Vivaldi, and H. Rodríguez Hontoria, ‘Syntactic methods for negation detection in radiology reports in Spanish’, Berlin, Germany, 2016, pp. 156–165, doi: 10.18653/v1/W16-2921.

[161] A. Cocos, T. Qian, C. Callison-Burch, and A. J. Masino, ‘Crowd control: Effectively utilizing unscreened crowd workers for biomedical data annotation’, *Journal of Biomedical Informatics*, vol. 69, pp. 86–92, May 2017, doi: 10.1016/j.jbi.2017.04.003.

[162] H. Chen, V. Gangaram, and G. Shih, ‘Developing a More Responsive Radiology Resident Dashboard’, *J Digit Imaging*, vol. 32, no. 1, pp. 81–90, Feb. 2019, doi: 10.1007/s10278-018-0123-6.

[163] Y. Zhang, D. Merck, E. B. Tsai, C. D. Manning, and C. P. Langlotz, ‘Optimizing the Factual Correctness of a Summary: A Study of Summarizing Radiology Reports’, *arXiv:1911.02541 [cs.CL]*, 2019, Accessed: Oct. 30, 2020. [Online]. Available: http://arxiv.org/abs/1911.02541.

[164] M. Yetisgen, P. Klassen, L. McCarthy, E. Pellicer, T. Payne, and M. Gunn, ‘Annotation of Clinically Important Follow-up Recommendations in Radiology Reports’, in *Proceedings of the Sixth International Workshop on Health Text Mining and Information Analysis*, Lisbon, Portugal, Sep. 2015, pp. 50–54, doi: 10.18653/v1/W15-2606.
